# Comparative Whey Proteome Profiling of Donkey Milk With Human and Cow Milk

**DOI:** 10.3389/fnut.2022.911454

**Published:** 2022-06-27

**Authors:** Xinhao Zhang, Guimiao Jiang, Chuanliang Ji, Zhaobin Fan, Shihao Ge, Haijing Li, Yantao Wang, Xin Lv, Fuwei Zhao

**Affiliations:** ^1^Department of Animal Pharmacy, College of Pharmacy, Heze University, Heze, China; ^2^National Engineering Research Center for Gelatin-Based TCM, Dong-E E-Jiao Co., Ltd., Liaocheng, China

**Keywords:** donkey milk, human milk, cow milk, whey, proteome

## Abstract

Donkey milk (DM), similar to human milk (HM) in chemical composition, has been suggested as the best potential hypoallergenic replacement diet for babies suffering from Cow milk (CM) protein allergy. In order to better understand DM protein, many studies based on proteomic have been performed. In this study, the label-free quantitative proteomic approach was conducted to quantitatively identify the differentially expressed whey proteins (DEPs) in DM vs. HM group and DM vs. CM group. In total, 241 and 365 DEPs were found in these two groups, respectively. Bioinformatics analysis of DEPs showed that the majority of DEPs participated in the lipoprotein metabolic process, regulation of cytokine production, chemical homeostasis, and catabolic process. The Kyoto Encyclopedia of Gene and Genomes (KEGG) pathways analysis found that these DEPs mainly participated in an antigen processing, complement, and coagulation cascades. These results may provide valuable information in the composition of milk whey proteins in DM, HM, and CM, especially for low abundant components, and expand our knowledge of different biological functions between DM and HM or CM.

## Introduction

Extensive studies on the nutritional properties and chemical composition of donkey milk (DM) have confirmed that it differs from milk from other mammals while being similar to human milk (HM) (1), mainly, in lactose, mineral content, fatty acid and protein composition (2).

For example, the total whey protein content of DM ranges between 0.49 and 0.80 g/100 g, very close to HM (0.68–0.83 g/100 g) (3). Studies have shown that DM can be used as a hypoallergenic dietary substitute for infants with cow milk (CM) protein allergy or intolerance (4, 5). DM is well tolerated at oral challenge by more than 80% of the children suffering from IgE-mediated cow's milk allergy (6). Because the cholesterol content of DM is very low, it has the function of preventing metabolic diseases and has immune regulation effect on the elderly (7). CM contains about 3% protein, among which whey protein has important biological activity and its composition is different in milk of different species, which also leads to changes in milk composition and biological function (8). For example, the major whey proteins in DM are α-lactalbumin (α-LA), β-lactoglobulin (β-LG) and lysozyme (LYS), but β-LG, which has been identified as the main allergen in CM does not occur in HM (9, 10). In the mare's milk three molecular types of α-LA have been isolated while only one genetic variant was found in DM (11). With the advances in the proteomics technology, more and more proteins in milk can be accurately detected, especially low abundance proteins. In recent years, investigations on DM whey proteome are on the rise and many whey proteins have been identified using proteomic approaches. Previous study has reported the characterization of the protein profile of the DM whey fraction by direct RP-HPLC/electrospray ionization (ESI)-MS analysis (12). Narrow pH (3.5–6) range two-dimensional gel electrophoresis (2-DE) coupled with mass spectrometry was applied to compare the whey proteins of DM, CM, and HM, with a particular focus on the major CM allergens (13). The HPLC-MS/MS-based proteomic method was used to analyze the differences of whey protein between DM and CM (14). Comparative analysis of whey proteins in donkey colostrum and mature milk was also performed using quantitative proteomics (15). Our previous study analyzed the difference in proteomic levels of DM whey proteins with different yield by label-free mass spectrometry (16). These studies provided useful information regarding the DM whey protein components; however, there are relatively few studies on the difference of whey protein between DM and breast milk based on quantitative proteomics. DM has been confirmed to be similar to HM and could be a better alternative to CM for infants (4, 17). However, most of the studies mainly focused on the major components, and the low abundant components in milk are emerging and found to be of great importance. The aim of this study was to describe the relative abundance of different proteins in whey between DM and HM or CM by using label-free quantification with LC/MS, a proteomic method that can identify the entire expression profile of protein samples with or without significant differences in abundance between the groups. The comprehensive analysis of these variations could gain a better understanding of the biological function and differences between DM and HM or CM, promote the utilization of DM as nutrition provider.

## Materials and Methods

### Sample Collection and Treatment

The HM samples were donated by 12 healthy lactating mothers between 6th and 8th month of lactation, with written informed consent stating that HM samples were going to be used for the research only. Twelve DM samples were collected from a local farm of Dezhou donkeys in Liaocheng City of Shandong province, China. About 12 CM samples were bulk milk obtained in a local farm with the permission of farm manager Liaocheng, China). The samples were collected and stored in an ultra-low temperature refrigerator at −80°C. The samples were thawed before use, mixed in four samples (in triplicate for each sample group) and centrifuged with 10,000 × g/min for 10 min to remove the cream layer. A mammalian protease inhibitor cocktail was added to each pooled sample, which was then depleted of casein by using a previously described method (18). Briefly, add 60 mM CaCl_2_ to the sample and adjust the pH to 4.3. Supernatant was collected by centrifugation at 4°C and 18,900 × g/min for 60 min.

### Protein Extraction and Digestion

The SDT buffer (2% SDS, 100 mM Tris/HCl pH 7.6, 0.1 M DTT) was used to dissociate the samples and extract the proteins. The protein concentration of protein lysate was determined by a BCA protein assay kit (Bio-Rad, USA), and proteins were trypsinized in accordance with the filter-aided sample prep (FASP) protein digestion protocol (19). The digested peptides of all samples were desalted on the C18 cartridges (Empore™ SPE C18 Cartridges, Sigma), centrifuged under vacuum conditions and reconstituted in 40 μl formic acid (0.1%).

### LC-MS/MS Analysis

In this study, the Q Exactive mass spectrometer coupled to Easy nLC (Proxeon Biosystems, Thermo Fisher Scientific) was used for MS experiments. In this procedure, 5 μg of the peptide was loaded onto the C18-reversed phase column (Thermo Scientific Easy Column, 10 cm long, 75 μm inner diameter, 3 μm resin) in buffer A (2% acetonitrile and 0.1% formic acid). The separation was performed with buffer B (80% acetonitrile and 0.1% formic acid) in a linear gradient for 120 min at a flow rate of 250 nL/min.

The MS data were collected with a data-dependent top 10 method by dynamically choosing the most abundant precursor ions from the survey scan (300–1,800 *m*/*z*) for higher energy collisional dissociation (HCD) fragmentation. The target value was determined based on predictive Automatic Gain Control (pAGC). The survey scans were acquired at a resolution of 70,000 at *m*/*z* 200 and dynamic exclusion duration of 25 s. Resolution of the HCD spectra was set to 17,500 at 200 *m*/*z* and an isolation width of 2 *m*/*z* (20). Normalized collision energy was 30 eV, and the under-fill ratio was defined as 0.1%. The instrument was run under the peptide recognition enablement mode.

### Sequence Database Searching and Protein Quantification

The MS data were analyzed using MaxQuant (version 1.3.0.5) and searched against the UniProtKB *Equus asinus* database (47,825 total entries, downloaded on Aug 12, 2019), uniprot *Bos taurus* (included 45,847 series, downloaded on May 7, 2019) and *homo sapiens* (included 20,422 series, downloaded on May 22, 2019). A mass accuracy tolerance of 6 ppm was set as the precursor mass window for the database search. The search used a trypsin/P enzymatic cleavage rule with a maximum of two missed cleavage sites and a mass tolerance of 20 ppm for fragment ions. The carbamidomethylation of cysteines were set as fixed modifications, whereas protein N-terminal acetylation and methionine oxidation were described as variable modifications for database searching. The threshold for the overall false discovery rate (FDR) for peptide and protein identification was set at 0.01. Protein quantification was performed using the (label-free quantification) LFQ algorithm implemented in the MaxQuant software. The LFQ values were log_2_ transformed and imputation for missing values using the Perseus software (width, 0.3; down-shift, 1.8). To identify the proteins with significantly different abundance between DM and CM or HM, a two-tailed and paired Student's *t*-test was performed.

### Functional Analysis of DEPs

The protein sequences of the selected DEPs were locally searched using BLAST to the NCBI database and Inter ProScan to find homolog sequences.Blast2GO program was used to map Gene ontology (GO) terms and annotate each sequence. Afterward, we performed online Kyoto Encyclopedia of Genes and Genomes (KEGG) pathway annotation of these DEPs and screened the target pathways following enrichment analysis. Quantified whey samples were performed for hierarchical clustering by “pheatmap” package in R (v4.0.2). David online tools (https://david.ncifcrf.gov/) were used to perform GO annotation and KEGG pathway Enrichment analyses of DEPs.

## Results

### Quantitative Overview of Identified Whey Proteins in Different Species

**Figure 1:**
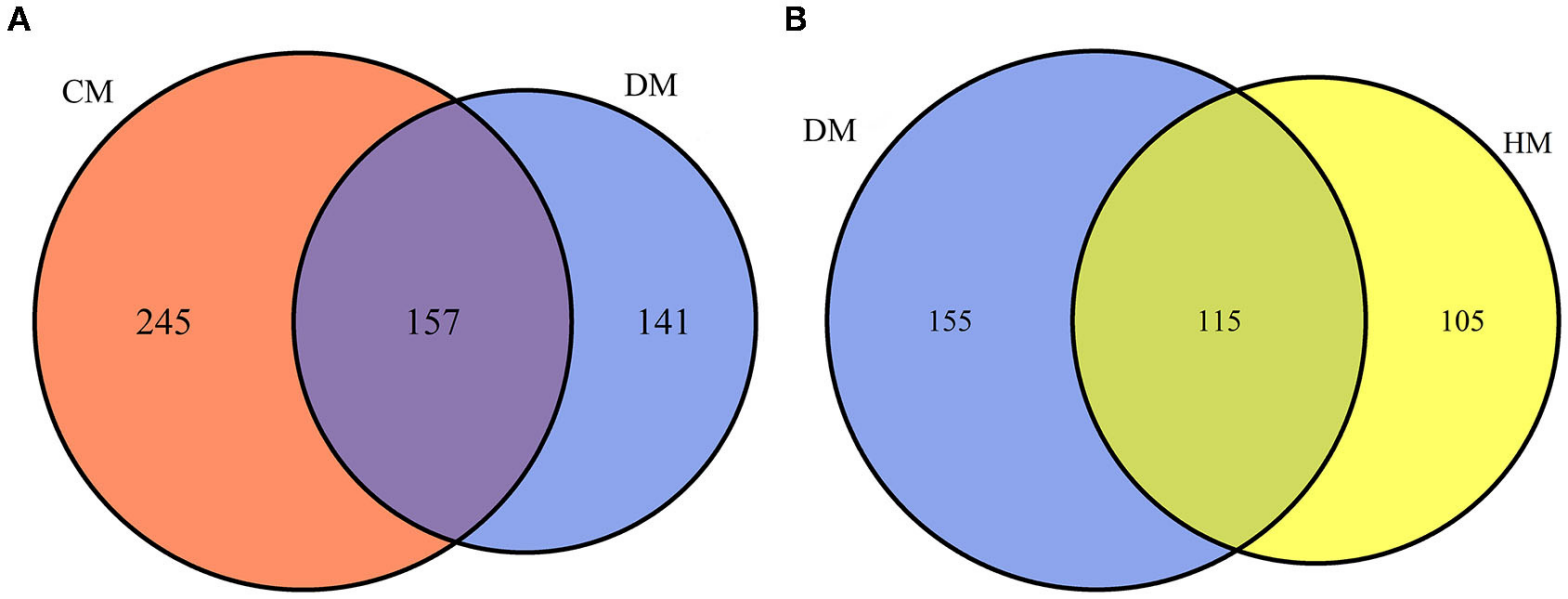
Venn diagram of proteins identified from DM vs. HM group and DM vs. CM group. **(A)** Number of proteins quantitatively identified in DM vs. CM group. **(B)** Number of proteins quantitatively identified in DM vs. HM group.

In this study, a total of 543 whey proteins were identified between DM and CM and 375 whey proteins were identified between DM and HM using a label-free proteomic approach (Figure 1). Compared with CM, 365 DEPs were identified, 138 of them were up-regulated and 227 of them were down-regulated in DM. Compared with HM, 241 DEPs were identified, of which 138 whey proteins were up-regulated and 103 were down-regulated in DM, respectively. The detailed information of DEPs was listed in Supplementary Table 1.

### Cluster Analysis

**Figure 2:**
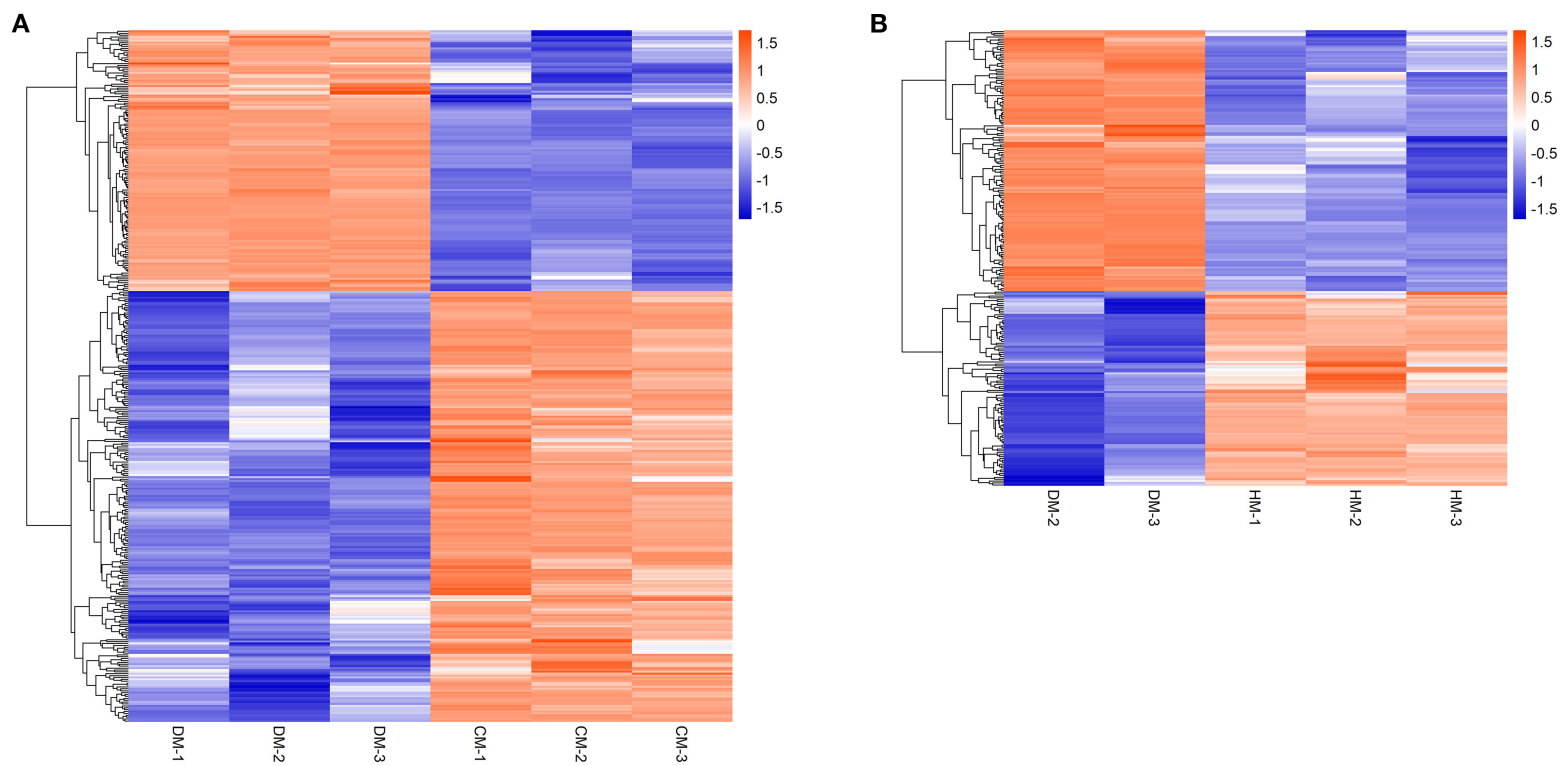
Hierarchical clustering of DEPs in DM vs. CM group **(A)** and DM vs. HM group **(B)**. The bar color represents a logarithmic scale from −1 to 1. Each column indicates a replicate experiment, and each row indicates a protein.

Hierarchical cluster analysis was used to analyze the DEPs to compare the expression patterns of whey proteins between DM and HM or CM (Figure 2). The upregulated whey proteins in DM compared with HM mainly included vitamin D binding protein, joining chain of multimeric IgA and IgM, polymeric immunoglobulin receptor, lysozyme, and the downregulated whey proteins were peptidyl-prolyl cis-trans isomerase, elongation factor 1-alpha and lipoprotein lipase. The upregulated whey proteins in DM compared with CM mainly included vitamin D binding protein, serum albumin, joining chain of multimeric IgA and IgM, polymeric immunoglobulin receptor, and the downregulated whey proteins were peptidyl-prolyl cis-trans isomerase, nucleobindin 2, and so on.

### GO Analysis of DEPs in DM Compared With HM and CM

**Figure 3:**
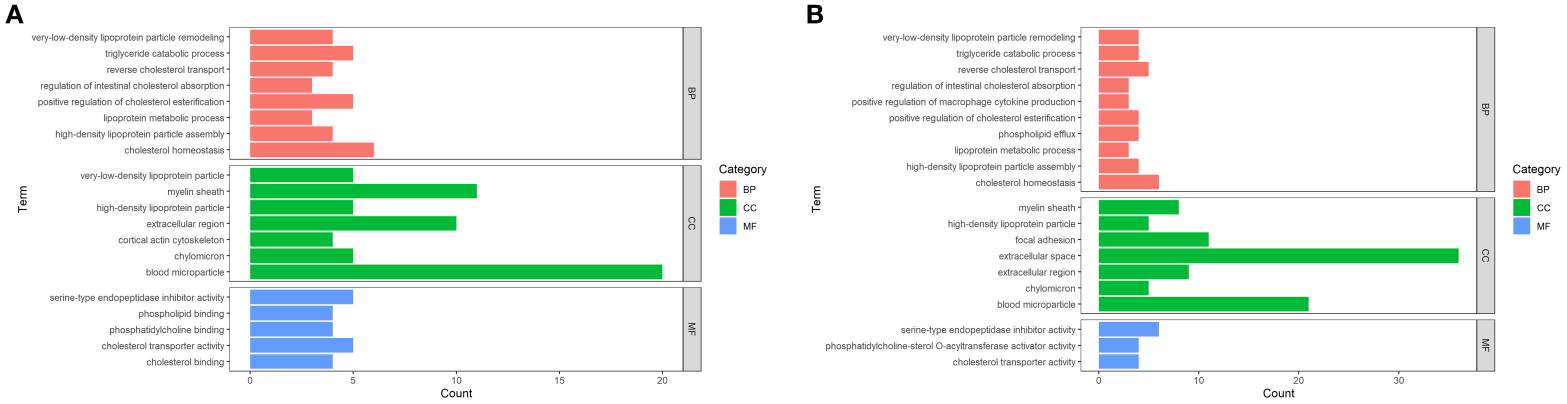
Enriched GO Terms of the DEPs in DM vs. CM group **(A)** and DM vs. HM group **(B)**. GO enrichment of DEPs on three categories. The length of the bar chart represents the count of proteins enriched to GO term.

According to the enrichment analysis, GO terms such as biological process (BP), cellular component (CC), and molecular function (MF) with FDR <0.05 were considered as statistically significant (Figure 3). In terms of MF, DEPs between DM and HM or CM were both primarily related to lipid binding and steroid binding. In the category of BP, DEPs in these two groups were mainly involved in the lipid metabolic process. Other major BP categories included regulation of cytokine production, chemical homeostasis and catabolic process. The detailed information on the GO terms of DEPs was listed in Supplementary Table 2.

### KEGG Pathway Analysis of DEPs in DM Compared With HM and CM

**Figure 4:**
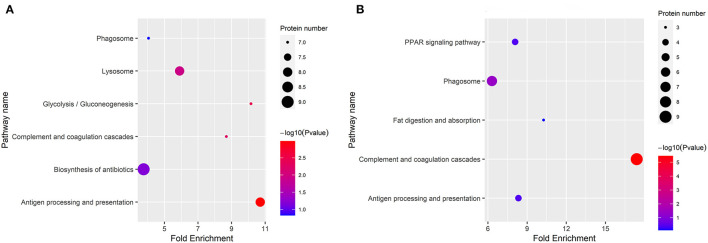
Enriched KEGG pathway analysis of the DEPs in DM vs. CM group **(A)** and DM vs. HM group **(B)**. The bubble size indicates the amount of protein enriched into the pathway. The color gradient corresponds to the magnitude of *p*-value. The gradient becomes red and indicates a small *p*-value.

The KEGG categories were determined using a hypergeometric statistical test. In Figure 4, the DEPs in DM vs. HM group and DM vs. CM group were mainly involved in complement and coagulation cascades, and antigen processing and presentation. The detailed information on the KEGG pathway enrichment of DEPs is listed in Supplementary Table 2.

## Discussion

In this study, we applied a label-free quantitative proteomics approach to compare the whey proteome profiling of DM with that of HM or CM. In total, 241 DEPs were found to be significantly different between DM and HM; 365 DEPs were significantly different between DM and CM. These DEPs were mainly involved in lipid metabolism, regulation of cytokine production, chemical homeostasis, and catabolism. These results may provide valuable information for studying the composition of whey proteins in DM, HM, and CM, especially for quantitative analysis of low abundance components, and expand our knowledge of their biological functions.

The DM was characterized by particularly high whey protein content, which was rich in lysozyme (21), α-La,β-Lg (22) and serum albumin (SA) (4). In our data, we also found these whey proteins in DM were significantly higher than HM and CM. For example, the amount of lysozyme in DM has proved to be higher with respect to that in CM and goat's milk (23). As a powerful antibacterial protein, lysozyme plays an important role in the intestinal immune response. DM lysozyme belongs to C-type calcium-binding lysozyme and could bind calcium ions which leads to more stable complex with an enhanced antimicrobial activity (24). β-Lg is the major whey protein in DM and CM, whereas in HM the β-Lg is absent (25–27). However, in present study β-Lg was identified in HM with low abundance.

According to the GO and KEGG pathway enrichment analysis, DEPs in DM vs. HM group and DM vs. CM group were all mainly involved in lipid binding, lipid metabolic process, and cholesterol pathway. Among these DEPs, ApoA1, ApoA2, ApoA4, ApoE, and ApoH in apolipoprotein family were significantly upregulated in DM. ApoA1 is the main protein component of high-density lipoprotein (HDL), which plays an important role in maintaining cholesterol homeostasis (28). ApoA2 may serve as a potential therapeutic target for atherosclerosis (29). ApoA1 has multiple beneficial functions, such as cardio protective, potent antioxidant and anti-inflammatory (30–33). In fat globule membrane (MFGM) of DM, ApoA1 was upregulated in colostrum compared with mature milk (34). Studies have shown that ApoA1 size in breast milk is directly related to embryo survival rate (35). The main function of ApoE is to transport cholesterol from peripheral tissues to the liver for metabolism (36). Some studies have linked ApoE to longevity and cognitive function in long-lived individuals (37). The concentration of ApoE in colostrum was higher than that in mature milk, indicating the importance of cholesterol in neonatal development (38, 39). It was suggested that high levels of cholesterol in breast milk may promote a healthier lipid profile in late adolescence through a mechanism unrelated to obesity, possibly with potential long-term benefits for cardiovascular health (40). In our study, DM provided higher levels of cholesterol transporters than HM and CM, which may contribute to the large amount of cholesterol in newborns. In addition to cholesterol transport function, recent work in both cell culture and in mice indicates that HDL (mainly ApoA1 and ApoA2) have anti-atherogenic effects and cause robust activation of endothelial nitric oxide (NO) synthase (28). ApoE deficiency in mice resulted in a profound susceptibility to atherosclerosis (41). Meanwhile, DM could induce human peripheral blood mononuclear cells (PBMCs) to release NO, which was very useful in the prevention of atherosclerosis (7). Therefore, we hypothesized that these upregulated Apos might contribute the effect to atherosclerosis prevention induced by DM.

Milk provides large amounts of bioactive components to the infants in the critical phase of immunological immaturity of the newborn, particularly for the immune system of mucous membranes. Breastfeeding protects infants against infections mainly *via* secretory IgA (SIgA). IgM, the second most abundant im-munoglobulin in human colostrum, are also important in protecting the mucosal surfaces of infants through its reaction with viruses and bacteria (42). Both dimeric IgA and pentameric IgM are transported across the epithelial cells into the milk by the polymeric Ig receptor (pIgR), expressed on the basolateral surface of mammary epithelial cells (43, 44). In addition to the heavy and light chains, dimeric IgA and pentameric IgM contain a small polypeptide known as the joining (J) chain, which plays an important role in the generation of secretory antibodies, because it provides them with the capacity to bind the pIgR (45). This peptide can be produced by immunocytes of all Ig isotypes, but it becomes incorporated only into IgA and pentameric IgM (46). In addition, J-chain expression may be a marker of B-cell cloning from mucosa-associated lymphoid tissues, since the production of polymeric IgA, IgG, or IgD-producing cells are positively correlated with J-chain (46, 47). In our data, J chain was significantly upregulated in DM vs. HM group (18.0-fold) and DM vs. CM group (3.3-fold) respectively. We speculated that this high level of J chain production might reflects the abundant IgA, IgM or other immunoglobulins in DM and contributes to explain the previous studies indicating that DM intake improves anti-inflammatory defenses in rats (48). Other abundant whey proteins related to the immune responses in DM included secreted phosphoprotein 1 (SPP1), complement C2, and complement C3. SPP1, present in significant amounts in breast milk (49), is a multifunctional protein involved in the cell-mediated immune responses and anti-inflammatory responses (50, 51). The studies have shown that the complement proteins help newborns to build up their natural immune systems (52). The existence of abundant immunological factors in DM can help the newborns to establish an immune system to resist microbial infection, adapt to the new environment and prevent diseases.

## Conclusion

In this study, a quantitative proteomic method was used to investigate the whey proteins proteome in DM, HM, and CM, which will help us to understand the nutritional composition across different species comprehensively. DEPs were mainly involved in the lipid metabolic process, regulation of cytokine production, chemical homeostasis, and catabolic process, which are of great significance to identify functional factors beneficial to infants. Our findings also provided a more in-depth reference for the dairy food industry and for the health of infants.

## Data Availability Statement

The raw data supporting the conclusions of this article will be made available by the authors, without undue reservation.

## Ethics Statement

The studies involving human participants were reviewed and approved by Institutional Animal Care and Use Committee of Heze University. The patients/participants provided their written informed consent to participate in this study.

## Author Contributions

FZ: study design, article review, quality assessment, and draft written. XZ: statistical analysis, draft written, and manuscript revise. GJ, HL, YW, CJ, XL, ZF, and SG: recruited samples and interpreted the data. All authors have read and approved the manuscript and ensure that this is the case.

## Funding

This study was supported by Doctoral Fund Project of Heze University (No. XY21BS37). The funding body has no role in the study design and data collection, analysis, interpretation of data, and in writing the manuscript.

## Conflict of Interest

XZ, GJ, CJ, HL, YW, and XL were employed by the company Dong-E E-Jiao Co., Ltd. The remaining authors declare that the research was conducted in the absence of any commercial or financial relationships that could be construed as a potential conflict of interest.

## Publisher's Note

All claims expressed in this article are solely those of the authors and do not necessarily represent those of their affiliated organizations, or those of the publisher, the editors and the reviewers. Any product that may be evaluated in this article, or claim that may be made by its manufacturer, is not guaranteed or endorsed by the publisher.

## Abbreviations

DM: donkeymilk
HM: humanmilk
CM: cowmilk
DEPs: differentially expressed whey proteins
α-La: α-lactalbumin
β-Lg: β-lactoglobulin
ApoA1: polipoprotein A1
MFGM: fat globule membrane
SPP1: secreted phosphoprotein 1.
