# Supplementary material for: Comparative Whey Proteome Profiling of Donkey Milk With Human and Cow Milk
Source: Front Nutr. 2022 Jun 27;9:911454. doi: 10.3389/fnut.2022.911454 (PMC9282231; doi:10.3389/fnut.2022.911454)
Supplement: Supplementary file 1 [file Data_Sheet_1.PDF]

1.Detailed information of the differentially expressed proteins (DM vs CM)

UniProt Accession	Protein Name	Coverage	Mol/Weight	LFQ intensity						-Log(p-value)	Log2(fold change)	Change
				DM-1	DM-2	DM-3	CM-1	CM-2	CM-3			
P08896	Alpha-lactalbumin B/C	42.3	14.25	0.95	0.90	0.89	-0.91	-0.89	-0.94	6.61	1.83	up
F7C0Y4	amyloid A protein	34.6	14.34	0.94	0.90	0.90	-0.92	-0.85	-0.96	6.16	1.82	up
F7BFV9		8.2	46.91	0.93	0.94	0.87	-0.90	-0.96	-0.88	6.10	1.82	up
A0A3Q2HQS1		0.6	188.40	0.89	0.98	0.87	-0.91	-0.93	-0.89	6.07	1.82	up
F6WI21	U2AF homology motif kinase 1	1.7	58.52	-0.86	-0.96	-0.91	0.91	0.87	0.95	6.01	-1.82	down
P13613	Beta-lactoglobulin-1	56.2	18.53	0.93	0.92	0.89	-0.84	-0.97	-0.93	5.93	1.82	up
F6RM73	Apolipoprotein A-II	18.5	17.40	0.95	0.88	0.90	-0.98	-0.89	-0.87	5.88	1.82	up
F7C450	2-HS glycoprotein	23.5	28.34	0.84	0.97	0.93	-0.88	-0.96	-0.89	5.60	1.82	up
A0A3Q2H3D5		9.1	24.05	-0.92	-0.84	-0.98	0.87	0.95	0.91	5.57	-1.82	down
A0A0A1E417	lambda light chain variable region (Fragment)	24.9	23.43	0.84	0.96	0.93	-0.84	-0.93	-0.96	5.49	1.82	up
A0A0A1E691	lambda light chain variable region (Fragment)	28.7	23.04	0.90	0.97	0.87	-0.83	-0.94	-0.97	5.43	1.82	up
P08334	Alpha-lactalbumin A	91.1	14.22	0.85	0.95	0.94	-0.92	-0.98	-0.83	5.40	1.82	up
P86273	Beta-casein	18.6	25.53	0.86	1.01	0.86	-0.88	-0.90	-0.95	5.39	1.82	up
P11375	Lysozyme C	62.8	14.69	0.87	0.95	0.92	-0.98	-0.93	-0.82	5.34	1.82	up
H9GZN9	Uncharacterized protein	31.4	56.92	0.86	0.96	0.91	-0.95	-0.96	-0.82	5.31	1.82	up
A0A3Q2HQW3	3-monooxygenase/tryptophan 5-monooxygenase activation protein beta	22.8	28.11	-0.90	-1.00	-0.84	0.97	0.86	0.91	5.30	-1.82	down
P19647	Beta-lactoglobulin-2	71.8	18.26	0.96	0.91	0.87	-1.00	-0.83	-0.91	5.29	1.82	up
A0A0A1E4I0	lambda light chain variable region (Fragment)	30.6	22.70	0.87	0.92	0.95	-0.83	-0.89	-1.01	5.24	1.82	up
F7B5P1	dipeptidase 2	13.3	52.85	-0.95	-0.92	-0.87	0.91	1.00	0.82	5.18	-1.82	down
F6YNH6		38.8	37.72	0.90	1.00	0.83	-0.89	-0.97	-0.87	5.18	1.82	up
A0A3Q2H6L1	Membrane cofactor protein	7.2	41.35	0.95	0.94	0.84	-0.95	-0.81	-0.97	5.09	1.82	up
A0A3Q2HNV2	Insulin-like growth factor-binding protein 2	28.2	34.82	-1.03	-0.83	-0.87	0.93	0.88	0.93	5.08	-1.82	down
F7BKE1	family F member 1	11	46.12	0.92	0.86	0.95	-0.82	-1.02	-0.90	5.06	1.82	up
A0A3Q2HYP3	phosphoprotein 1	45.3	34.98	0.95	0.89	0.89	-0.79	-0.96	-0.98	5.02	1.82	up
A0A3Q2I0A2	Fibroblast growth factor-binding protein 1	17	24.30	0.92	0.86	0.95	-0.86	-0.85	-1.03	5.01	1.82	up
A0A3Q1MJJ4	Apolipoprotein A-I	10	28.74	-0.86	-1.00	-0.87	0.91	0.83	0.99	5.01	-1.82	down
H9GZT5	Uncharacterized protein	33.2	43.03	0.89	0.90	0.94	-0.82	-0.88	-1.03	4.98	1.82	up
A0A3Q2GWN9	Beta-1 metal-binding globulin	47.5	78.05	0.91	0.91	0.91	-1.04	-0.83	-0.86	4.94	1.82	up

A0A3Q2HN20	albumin	77.9	66.74	0.91	0.93	0.89	-0.95	-0.78	-1.00	4.93	1.82	up
I3RM62	2-phospho-D-glycerate hydro-lyase	27.6	47.14	-0.97	-0.96	-0.81	1.00	0.89	0.84	4.86	-1.82	down
Q95M34	gamma 1 heavy chain constant region (Fragment)	45.4	37.44	0.89	0.96	0.87	-0.90	-1.03	-0.80	4.85	1.82	up
F7C7V8	Semaphorin 7A	25.6	74.53	0.91	0.95	0.87	-1.01	-0.77	-0.94	4.77	1.82	up
C7E3N6	fatty acid-binding protein	35.1	14.94	0.80	1.02	0.91	-0.92	-0.97	-0.84	4.76	1.82	up
F6ZI35	Histidine rich glycoprotein	3.9	57.93	0.88	1.02	0.83	-0.89	-0.83	-1.01	4.76	1.82	up
B5BV04	Alpha-1-antitrypsin	21.6	46.86	1.02	0.88	0.83	-0.82	-0.98	-0.94	4.74	1.82	up
F6U9Q8	nucleotide binding domain containing 2	1.3	61.80	0.79	1.02	0.92	-0.89	-0.86	-0.98	4.74	1.82	up
F6PKE1	Inhibitor of carbonic anhydrase	10	80.15	0.75	1.02	0.96	-0.91	-0.89	-0.93	4.65	1.82	up
H9GZV1	Uncharacterized protein	26.5	40.64	0.92	0.92	0.89	-0.98	-1.00	-0.75	4.65	1.82	up
H9GZU8	Uncharacterized protein	36.9	48.92	1.01	0.82	0.89	-0.88	-1.02	-0.83	4.64	1.82	up
Q3ZBH8	ribosomal protein S20	19.3	13.37	-0.94	-0.85	-0.94	1.06	0.84	0.83	4.62	-1.82	down
A0A3Q2ID55	carrier family 4 member 9	2.1	95.76	0.86	0.90	0.96	-0.88	-1.06	-0.79	4.54	1.82	up
A0A3Q2L0G4	domain-containing protein	3.3	48.26	0.98	0.78	0.97	-0.80	-0.93	-1.00	4.54	1.82	up
E1BCU6	Transcobalamin 1	2.1	47.26	-1.07	-0.84	-0.81	0.92	0.86	0.94	4.54	-1.82	down
Q6X9W5	amyloid A protein	34.2	13.28	0.96	0.88	0.89	-0.85	-1.07	-0.81	4.53	1.82	up
O97966	Transferrin	37.7	6.88	0.83	0.96	0.94	-1.00	-0.97	-0.76	4.52	1.82	up
F7APU2	Complement factor I	14.3	70.54	0.85	0.94	0.94	-0.77	-1.06	-0.89	4.47	1.82	up
U3LV13	Alpha-amylase	20.7	57.38	-0.99	-0.74	-0.99	0.97	0.86	0.89	4.44	-1.82	down
F6WR95	oxidase	8.6	82.98	-1.09	-0.83	-0.81	0.90	0.94	0.88	4.40	-1.82	down
F6ZBH7	nucleotide exchange factor	6.5	52.35	-0.88	-1.06	-0.79	0.81	0.95	0.96	4.36	-1.82	down
F6UL68	Transthyretin	19.4	15.50	0.88	1.08	0.76	-0.93	-0.92	-0.87	4.35	1.82	up
F7D837	alpha 1	12.4	100.10	-0.83	-1.03	-0.87	1.00	0.97	0.76	4.32	-1.82	down
B7VGF9	Alpha-S2-casein	69.9	27.70	0.94	1.05	0.73	-0.93	-0.87	-0.93	4.32	1.82	up
A0A3Q2KT61	4,5-bisphosphate phosphodiesterase gamma	1.1	144.43	0.88	0.93	0.92	-0.80	-0.82	-1.10	4.30	1.82	up
Q95114	Lactadherin	5.4	47.41	-0.75	-1.04	-0.94	0.99	0.92	0.81	4.29	-1.82	down
A0A3Q2GVU8	Annexin	8.8	38.60	-0.91	-1.07	-0.74	0.91	0.96	0.85	4.25	-1.81	down
K9K2D2	dehydrogenase	21.9	36.40	-0.85	-0.78	-1.08	0.98	0.91	0.83	4.24	-1.81	down
A0A3Q2GS25	ester hydrolase	15.4	65.14	-0.86	-0.76	-1.10	0.94	0.90	0.88	4.24	-1.81	down
A0A3Q2H4E3	carrier family 34 member 2	6.4	79.24	0.77	0.95	1.00	-1.06	-0.84	-0.82	4.24	1.81	up
F6VJR6	Alpha-1B-glycoprotein	28	44.90	0.93	0.90	0.90	-0.72	-1.07	-0.94	4.24	1.81	up
F7CSL8	domain-containing protein	15	46.87	0.92	0.84	0.96	-0.77	-0.87	-1.09	4.24	1.81	up
A0A2K9YV04	Lysozyme	12.8	16.74	-0.73	-0.96	-1.03	0.98	0.82	0.92	4.24	-1.81	down
A0A3Q2LNC9	intracellular cholesterol transporter 2	31.4	16.50	0.89	0.94	0.89	-0.82	-0.79	-1.11	4.23	1.81	up
A0A0A1E6I7	lambda light chain variable region (Fragment)	26.7	23.08	0.98	0.95	0.79	-1.06	-0.77	-0.89	4.23	1.81	up

F6W4R2	Angiotensin 1-10	6.5	51.78	0.97	1.01	0.74	-1.01	-0.92	-0.79	4.18	1.81	up
F6T7A1	domain containing 1	16.5	23.31	-0.80	-0.83	-1.08	0.90	0.81	1.01	4.15	-1.81	down
A0A3Q2I2H1	domain-containing protein	38	10.65	0.90	0.93	0.89	-0.70	-0.96	-1.06	4.14	1.81	up
P02758	Beta-lactoglobulin-1	62.2	20.34	0.91	0.92	0.89	-0.76	-0.84	-1.12	4.13	1.81	up
F6Z3A6	Transporter	1.2	67.00	-1.02	-0.98	-0.72	0.88	0.84	1.00	4.13	-1.81	down
P11376	C, milk isozyme	74.4	14.65	0.90	0.91	0.90	-1.07	-0.70	-0.95	4.11	1.81	up
A0A3Q2IDD2	C3/C5 convertase	20.2	85.51	0.92	0.80	1.00	-1.07	-0.76	-0.89	4.11	1.81	up
F1MS05	aconitate hydratase	6	98.23	-1.03	-0.73	-0.96	0.92	1.01	0.78	4.07	-1.81	down
A8YXY3	Selenoprotein F	9.9	17.97	0.97	0.94	0.80	-1.01	-1.00	-0.71	4.05	1.81	up
Q6X9X1	Beta-2-microglobulin	7.4	10.98	0.95	0.70	1.06	-0.95	-0.82	-0.94	4.02	1.81	up
F6VUW2	Cathepsin S	21.7	37.32	0.85	0.94	0.93	-0.78	-0.81	-1.13	3.99	1.81	up
F7BN14	IFI30 lysosomal thiol reductase	15.9	27.41	0.87	0.99	0.85	-0.94	-0.70	-1.08	3.98	1.81	up
K9KFG5	protein subfamily C member 3-like protein	15.4	41.71	0.91	0.93	0.87	-0.68	-1.08	-0.95	3.97	1.81	up
G3X8D7	peroxidase	5.4	25.34	-0.86	-1.13	-0.72	0.88	0.97	0.87	3.92	-1.81	down
F6WG98	Transporter	1.7	71.93	0.82	0.83	1.06	-0.86	-0.77	-1.08	3.92	1.81	up
F6V881	Vitronectin	8.5	53.80	-0.87	-0.94	-0.90	1.06	0.67	0.99	3.90	-1.81	down
P28546	Alpha-lactalbumin	91.1	14.22	0.90	0.92	0.89	-0.65	-1.04	-1.02	3.87	1.81	up
A0A3S5ZPW7	dehydrogenase/oxidase	8.2	142.33	-1.12	-0.75	-0.84	0.79	0.95	0.97	3.87	-1.81	down
F7DXM5	domain-containing protein	15.7	46.76	0.95	0.90	0.86	-0.66	-1.04	-1.02	3.86	1.81	up
F6PXV9	oxide synthase	0.9	141.61	-0.87	-0.78	-1.06	1.09	0.80	0.82	3.86	-1.81	down
A0A3Q2I560	cotransporter	12.7	86.31	0.84	0.86	1.01	-1.02	-0.67	-1.02	3.85	1.81	up
Q5E956	isomerase	32.9	26.69	-1.06	-0.92	-0.73	1.02	0.94	0.74	3.84	-1.81	down
P86272	Alpha-S1-casein	30.2	24.41	0.83	1.06	0.82	-1.07	-0.92	-0.72	3.83	1.81	up
F7CL92	transaminase 1	18.5	78.86	-0.71	-0.94	-1.06	1.04	0.89	0.78	3.83	-1.81	down
F7ASU6	Selenium binding protein 1	10.6	52.61	-1.10	-0.72	-0.89	0.92	1.02	0.77	3.82	-1.81	down
A0A3Q2HIU8	fragment of IgA and IgM receptor	2.2	53.54	0.93	0.74	1.05	-0.89	-0.75	-1.07	3.81	1.81	up
A0A3Q1M4B8	Serine peptidase inhibitor, Kunitz type 1	5.9	56.98	-1.14	-0.89	-0.69	0.92	0.87	0.92	3.80	-1.81	down
C0HJR4	Lactadherin	100	2.60	0.82	0.86	1.02	-0.70	-0.91	-1.10	3.80	1.81	up
A6QNZ7	Keratin 10	16.3	54.85	-1.11	-0.70	-0.90	0.80	0.95	0.96	3.80	-1.81	down
Q9BDZ7	TPR motif protein	2.6	30.43	-0.83	-0.78	-1.10	0.97	0.73	1.01	3.79	-1.81	down
Q3ZCI4	dehydrogenase, decarboxylating	9.3	53.08	-0.90	-0.71	-1.11	0.95	0.98	0.78	3.78	-1.81	down
Q08E11	cis-trans isomerase C	6.1	22.81	-0.95	-0.77	-0.99	0.95	1.07	0.69	3.78	-1.81	down
F6XSF7	C4a anaphylatoxin	13.7	174.06	-0.67	-0.96	-1.08	0.94	0.97	0.80	3.78	-1.81	down
A0A3Q1N756	protein kinase kinase kinase 4	1.9	151.70	-1.02	-0.67	-1.02	0.91	1.00	0.80	3.78	-1.81	down
B1PLB8	casein	43.9	14.90	0.86	1.02	0.83	-0.97	-0.67	-1.07	3.77	1.81	up
Q5E9B7	intracellular channel protein 1	26.1	26.99	-0.82	-0.87	-1.01	1.00	1.05	0.66	3.75	-1.81	down
A0A3Q1LQJ3	Secretagogin	6	30.89	0.86	0.93	0.92	-0.72	-0.83	-1.16	3.74	1.81	up
A0A3Q2H536	antigen	4.5	66.30	0.84	0.96	0.91	-0.99	-0.64	-1.07	3.71	1.80	up

A0A3Q1LT19	Ig-like domain-containing protein	6.2	13.87	-1.15	-0.82	-0.73	0.81	0.99	0.91	3.70	-1.80	down
A0A3Q2I5T2	domain-containing protein	10.6	15.20	0.94	0.70	1.07	-0.73	-1.00	-0.98	3.70	1.80	up
F6XEB4	3-monooxygenase/tryptophan 5-monooxygenase activation protein zeta	40.1	25.93	-0.96	-0.91	-0.83	1.12	0.93	0.66	3.70	-1.80	down
Q9TS52	differentiation-related protein (Fragments)	11.1	14.78	-1.17	-0.77	-0.77	0.94	0.80	0.97	3.65	-1.80	down
F6Z2L5	Apolipoprotein E	53.4	30.33	0.96	0.84	0.90	-0.94	-1.13	-0.64	3.61	1.80	up
F6U1J0	Complement factor H	4.4	134.70	0.86	0.94	0.91	-0.62	-1.08	-1.01	3.61	1.80	up
C3W972	s1 casein	28.3	25.28	0.85	1.16	0.69	-0.98	-0.79	-0.94	3.60	1.80	up
F5CEP2	fat globule-EGF factor 8 splice variant	54.1	43.31	0.85	0.82	1.04	-1.15	-0.72	-0.83	3.59	1.80	up
Q861V5	cis-trans isomerase	51	16.93	-0.74	-0.93	-1.03	0.91	1.10	0.68	3.58	-1.80	down
P35747	albumin	72	68.60	0.92	0.96	0.82	-0.61	-1.02	-1.06	3.57	1.80	up
F6YYP6	domain-containing protein	34.3	11.19	1.01	0.83	0.86	-0.95	-0.64	-1.11	3.56	1.80	up
A0A3Q2GXX9	22 C20orf96 homolog	2	40.76	0.71	0.92	1.07	-0.75	-0.84	-1.11	3.56	1.80	up
F7DY67	nucleoside phosphorylase	13.1	31.93	-0.95	-0.75	-1.00	1.10	0.96	0.64	3.49	-1.80	down
C1L3G3	s2 casein B	20	18.36	0.81	1.17	0.71	-0.77	-0.92	-1.01	3.49	1.80	up
A0A3Q2KRR2	CD55 molecule	13.9	92.46	0.88	0.93	0.88	-0.73	-0.75	-1.21	3.48	1.80	up
A0A3Q1LHW2	repeat domain 42	2.7	57.18	-1.00	-0.60	-1.10	0.82	0.92	0.95	3.47	-1.80	down
F6Y0D9	Cadherin-1	2.2	99.55	1.11	0.83	0.76	-1.13	-0.78	-0.79	3.47	1.80	up
F7DK89	translationally-controlled 1	15.7	19.60	-1.10	-0.70	-0.90	0.94	1.06	0.70	3.47	-1.80	down
A0A3Q2H839	Ig-like domain-containing protein	12.3	11.53	-0.72	-0.79	-1.18	0.87	0.79	1.03	3.46	-1.80	down
F7C1X7	exchange regulatory cofactor NHE-RF	6.3	39.56	-0.98	-0.73	-0.98	1.00	1.07	0.62	3.41	-1.80	down
A0A3Q1LRC3	protein	0.4	180.57	0.86	0.94	0.89	-0.90	-0.62	-1.18	3.41	1.80	up
Q862H8	to 40S ribosomal protein SA (P40)	17.5	18.61	-0.88	-1.08	-0.73	1.14	0.86	0.70	3.41	-1.80	down
A0A3Q2KY85	H	16.7	32.20	0.80	0.80	1.09	-0.78	-0.75	-1.17	3.40	1.80	up
A0A3Q1MFJ2	reductase	4.3	24.69	-0.64	-1.12	-0.93	1.08	0.82	0.79	3.36	-1.79	down
A0A3Q2HM99	WAP four-disulfide core domain protein 2	13.5	16.39	1.03	0.64	1.02	-0.86	-0.73	-1.11	3.36	1.79	up
F7D3H6	like proto-oncogene B	6.8	23.45	-0.81	-1.20	-0.68	1.01	0.88	0.80	3.35	-1.79	down
A0A3Q2H2S0	pathogenesis related 2	10.2	14.15	-1.16	-0.63	-0.90	0.76	0.95	0.98	3.34	-1.79	down
F6UZK9	lebercilin like	2.8	76.39	0.66	1.01	1.02	-0.90	-0.68	-1.11	3.34	1.79	up
F7B320	Dihydropyrimidinase-related protein 2	17.3	62.29	-0.60	-0.97	-1.11	1.04	0.86	0.79	3.34	-1.79	down
F7C603	phosphodiesterase acid like 3B	4	60.54	0.86	1.15	0.68	-1.10	-0.82	-0.77	3.33	1.79	up
A0A3Q2HT57	domain-containing protein	16.5	29.63	1.02	0.85	0.82	-1.11	-1.00	-0.59	3.33	1.79	up

P15467	Ribonuclease 4	34.5	13.74	-0.95	-1.16	-0.58	0.96	0.86	0.87	3.32	-1.79	down
A0A3Q2L2R4	domain-containing protein	4.9	20.72	0.94	0.93	0.82	-1.23	-0.67	-0.79	3.32	1.79	up
F6Z5E1	B	1.8	473.89	-1.19	-0.70	-0.80	0.77	1.05	0.87	3.31	-1.79	down
F7AAK7	gamma 1	58.4	41.79	-1.02	-0.97	-0.70	1.09	0.99	0.61	3.26	-1.79	down
A1XED1	RPL7	10.3	14.59	-0.75	-1.18	-0.76	1.07	0.93	0.69	3.24	-1.79	down
F1MXP8	Prosaposin	9.7	58.07	-1.23	-0.82	-0.63	0.82	0.92	0.94	3.22	-1.79	down
F6S3Y7	S100	11.4	8.96	0.67	0.97	1.04	-1.09	-0.62	-0.97	3.22	1.79	up
F6Z4J4	GDP dissociation inhibitor	32.1	50.42	-1.23	-0.81	-0.64	0.84	1.00	0.84	3.20	-1.79	down
A0A3Q1MHX8	RAB2A	27.1	22.47	-1.14	-0.65	-0.90	1.06	0.96	0.67	3.20	-1.79	down
A0A0A1E464	lambda light chain variable region	19.6	23.57	0.89	0.99	0.81	-1.20	-0.59	-0.89	3.20	1.79	up
A0A3Q2H4W0	domain containing 110	1.6	95.76	-1.26	-0.74	-0.68	0.89	0.95	0.84	3.18	-1.79	down
F7C0U4	domain-containing protein	6.1	40.90	-0.60	-0.90	-1.18	1.04	0.87	0.76	3.17	-1.79	down
Q58D84	Follistatin-related protein 1	19.2	34.86	-0.61	-0.85	-1.22	0.79	0.88	1.01	3.14	-1.79	down
Q2KIC8	Transmembrane protein 100	8.2	14.34	1.06	0.81	0.81	-1.13	-0.98	-0.56	3.13	1.79	up
F7CZW9	family G member 1	1.9	62.28	0.98	0.72	0.98	-0.95	-1.16	-0.57	3.13	1.79	up
F6T7K7	Transcobalamin 1	23.8	48.27	0.81	0.89	0.98	-1.24	-0.61	-0.82	3.13	1.78	up
F7CL80	actin depolymerizing factor	25.6	18.37	-0.90	-1.18	-0.59	1.03	0.92	0.72	3.12	-1.78	down
H9GZL8	acidic ribosomal protein P2	50.4	11.69	-0.75	-0.71	-1.22	0.89	1.08	0.71	3.12	-1.78	down
Q3ZC84	non-specific dipeptidase	13.5	52.66	-1.10	-0.57	-1.01	0.97	1.02	0.68	3.09	-1.78	down
A0A3Q2KUR5	receptor C-type 1	14.9	165.48	-0.79	-1.27	-0.61	0.89	0.88	0.90	3.09	-1.78	down
A0A3Q2HUS7	domain-containing protein	8.4	22.93	1.13	0.74	0.81	-1.00	-1.09	-0.58	3.08	1.78	up
F7BPX8	Cathepsin L1	36.2	37.40	0.91	0.90	0.87	-0.70	-0.69	-1.29	3.07	1.78	up
F6VBP9	Apolipoprotein E	15.2	34.97	0.80	1.02	0.85	-1.12	-0.52	-1.03	3.07	1.78	up
Q6VCR7	Transketolase	10.5	22.44	-0.95	-0.55	-1.18	1.00	0.74	0.94	3.06	-1.78	down
A0A3Q2HNJ8	protein	16.3	139.94	-0.92	-0.53	-1.22	0.92	0.90	0.85	3.05	-1.78	down
F6TZS9	Triosephosphate isomerase	24	29.93	-1.08	-0.51	-1.08	1.02	0.87	0.78	3.04	-1.78	down
F7D1R1	kinase	32.9	44.63	-1.17	-0.96	-0.55	1.06	0.86	0.75	3.02	-1.78	down
F7CU94	Lysozyme	4.7	16.72	-0.91	-1.23	-0.53	0.85	0.93	0.89	3.01	-1.78	down
A0A140T896	hydroxymethyltransferase	6.6	52.97	-0.55	-1.05	-1.07	1.10	0.86	0.71	3.01	-1.78	down
A0A3Q2IB08	translation elongation factor 2	13.9	95.38	-1.13	-0.68	-0.86	1.19	0.84	0.64	3.01	-1.78	down
F6W1N4	immunoglobulin receptor	31.9	83.00	0.88	0.93	0.86	-1.15	-0.48	-1.04	2.99	1.78	up
F6Z8W0	repeat domain 1	13.4	66.24	-1.19	-0.91	-0.56	1.04	0.93	0.70	2.99	-1.78	down
F6VB94	growth factor beta induced	28.6	74.31	1.19	0.69	0.78	-1.17	-0.73	-0.77	2.99	1.78	up
F1MPL4	diphosphate kinase B	23.7	12.79	-1.01	-1.14	-0.51	0.91	1.02	0.74	2.99	-1.78	down
F7B0S3	Milk fat globule EGF and factor V	41.6	54.87	0.79	0.85	1.02	-1.09	-1.08	-0.49	2.97	1.78	up
A0A3Q2I7B9	receptor, JUNO	6.2	20.56	-0.51	-1.20	-0.95	0.84	0.99	0.83	2.97	-1.78	down
Q9GKX7	shock protein HSP 90-alpha	28.1	84.77	-1.29	-0.62	-0.75	0.96	0.89	0.81	2.95	-1.78	down
A0A1L3G6H3	inhibitor of metalloproteinase 1	5.3	23.03	-1.29	-0.76	-0.61	0.95	0.92	0.79	2.94	-1.77	down
H9H007	dehydrogenase	10.6	99.01	-1.00	-0.89	-0.77	1.13	1.05	0.48	2.93	-1.77	down

E9RHW1	shock 27kDa protein 1	13.4	22.39	-0.67	-1.16	-0.82	1.20	0.80	0.66	2.92	-1.77	down
A0A3Q2H4G0	domain-containing protein	3.8	31.82	-1.00	-0.49	-1.17	0.80	1.04	0.82	2.91	-1.77	down
C0LSL0	fatty acid-binding protein	34.6	14.79	-0.78	-1.05	-0.82	1.19	0.50	0.96	2.89	-1.77	down
K9KAA4	protein sorting-associated protein 13C-like protein	3.1	36.03	-0.98	-1.19	-0.48	0.93	0.96	0.76	2.88	-1.77	down
K9KFW7	FAM76B-like protein	9.1	10.22	0.70	1.20	0.75	-1.18	-0.63	-0.84	2.85	1.77	up
A0A3Q2HSB3	Lactoperoxidase	2.6	78.11	-0.64	-0.72	-1.29	1.08	0.78	0.79	2.83	-1.77	down
F6VET1	reductase family 1 member D1	2.8	37.52	-0.59	-1.32	-0.74	0.93	0.91	0.81	2.83	-1.77	down
A1XEA6	muscle and non-muscle myosin alkali light chain peptide 6	52.7	6.07	-0.49	-1.17	-0.99	0.86	1.08	0.71	2.82	-1.77	down
F1MP21	Alpha-amylase	14.7	57.04	-0.69	-0.70	-1.25	1.16	0.80	0.68	2.77	-1.76	down
A0A3Q2HQX4	member RAS oncogene family	17	22.70	-0.88	-0.49	-1.27	0.77	1.01	0.87	2.77	-1.76	down
Q2KIM0	alpha-L-fucosidase	11.5	54.09	-1.24	-0.57	-0.83	0.79	1.15	0.71	2.76	-1.76	down
F1MJ80	phosphoribosyltransferase	20.4	53.67	-0.44	-1.22	-0.98	0.96	0.90	0.78	2.76	-1.76	down
P18203	cis-trans isomerase FKBP1A	25	11.91	-1.12	-0.43	-1.10	1.02	0.85	0.78	2.75	-1.76	down
A0A3Q2HRM6	protein subunit alpha	6.3	18.03	-1.13	-0.98	-0.53	1.19	0.71	0.75	2.75	-1.76	down
A0A3Q2LNJ1		10	91.90	-0.88	-0.68	-1.08	1.30	0.66	0.69	2.75	-1.76	down
A0A3Q1M0L3	initiation factor 4A-II	6.6	41.96	-0.50	-0.91	-1.23	1.10	0.83	0.71	2.74	-1.76	down
Q56JX8	ribosomal protein S13	6.6	17.22	-1.23	-0.46	-0.95	1.04	0.86	0.75	2.72	-1.76	down
F7BFT1	Peroxiredoxin 2	18.2	21.93	-1.14	-1.05	-0.45	0.95	1.03	0.66	2.72	-1.76	down
A0A3Q1LVA9	acid synthase	5.2	269.91	-1.32	-0.54	-0.78	1.03	0.84	0.76	2.71	-1.76	down
A0A3Q2KP77	Defensin beta 1	11.8	7.38	1.11	0.78	0.75	-0.47	-0.97	-1.20	2.71	1.76	up
F6VF11	Chordin like 2	25	46.35	-0.96	-0.99	-0.68	1.14	0.43	1.06	2.70	-1.76	down
P00727	aminopeptidase	14.1	56.29	-1.26	-0.45	-0.93	1.02	0.85	0.77	2.69	-1.76	down
F6W2Y1	gamma chain	8	49.80	0.52	0.84	1.28	-0.90	-1.07	-0.67	2.68	1.76	up
F6S0P5	ribosylation factor 4	27.2	20.53	0.52	0.86	1.26	-0.77	-0.73	-1.13	2.68	1.76	up
F6RZ27	Apolipoprotein E	10.8	43.25	0.74	0.96	0.94	-0.42	-0.98	-1.23	2.66	1.76	up
Q865P6	Beta-defensin 1	17.2	6.88	1.20	0.65	0.78	-0.86	-0.55	-1.22	2.65	1.75	up
Q0VBZ9	MARCKS-related protein	14.1	19.83	-1.36	-0.77	-0.50	0.88	0.96	0.78	2.61	-1.75	down
F6TVZ7	divalent cation tolerance homolog	13.1	18.79	1.33	0.51	0.78	-0.96	-0.70	-0.98	2.61	1.75	up
F1MH40	Uncharacterized protein	6.7	26.33	-1.07	-1.19	-0.37	0.86	0.86	0.91	2.61	-1.75	down
F6YZK3	domain-containing protein	8.5	11.71	0.87	1.12	0.64	-1.05	-1.13	-0.44	2.61	1.75	up
A0A3Q2LFU0	inhibitor heavy chain 1	2.6	96.76	0.92	0.80	0.90	-0.68	-1.38	-0.56	2.60	1.75	up
P60712	cytoplasmic 1	58.4	41.74	-1.02	-1.05	-0.56	1.04	1.12	0.47	2.60	-1.75	down
A0A3Q2LSE8	D interacting substrate 220	0.6	188.76	-0.69	-0.80	-1.14	1.18	1.00	0.45	2.60	-1.75	down
F6TQR2	Calreticulin	8.7	47.55	-0.86	-0.94	-0.82	1.39	0.66	0.58	2.59	-1.75	down
F7CTF0	hormone-related protein	15.3	20.43	-0.95	-0.43	-1.24	0.96	0.64	1.03	2.57	-1.75	down
A0A3Q2HTG2	alpha chain	6	77.40	0.77	0.62	1.23	-1.22	-0.84	-0.57	2.56	1.75	up
G3N0M6	CIDE-N domain-containing protein	6.2	23.73	-0.75	-1.38	-0.49	0.78	0.94	0.90	2.55	-1.75	down

Q2KIW9	UMP-CMP kinase	16.8	22.28	-0.96	-0.44	-1.22	1.14	0.81	0.67	2.55	-1.75	down
B3IVM1	Pyruvate kinase	8.9	57.83	-1.35	-0.49	-0.78	0.94	0.98	0.70	2.55	-1.75	down
F7DXG8	Cofilin	63.3	18.50	-0.69	-1.19	-0.74	1.24	0.89	0.49	2.54	-1.75	down
A0A3Q1M2P9	excision repair protein RAD23 homolog A	4.7	39.54	-0.36	-1.17	-1.08	0.98	0.94	0.70	2.53	-1.74	down
A0A3Q1M4K3	Ubiquitin-like domain-containing protein	44.2	8.63	-1.18	-0.59	-0.85	1.15	1.00	0.46	2.52	-1.74	down
F7E2D1	Actin-depolymerizing factor	22.1	80.74	-0.90	-1.27	-0.44	1.10	0.88	0.64	2.51	-1.74	down
A0A3Q1MQZ2	synthase	7.6	119.75	-1.10	-0.40	-1.11	1.07	0.96	0.58	2.48	-1.74	down
Q8WNR8	Perilipin	10.7	45.25	-0.37	-1.32	-0.92	0.80	0.85	0.96	2.47	-1.74	down
F6SP02	3-monooxygenase/tryptophan 5-monooxygenase activation protein theta	24.1	27.76	-1.37	-0.78	-0.46	0.96	0.95	0.70	2.46	-1.74	down
Q1ZYP1	Nck-associated protein	0.6	127.30	0.64	1.20	0.76	-1.25	-0.48	-0.88	2.46	1.74	up
K9KDP8	Peroxiredoxin-6-like protein	18.8	21.53	-0.96	-0.36	-1.29	0.98	0.90	0.72	2.45	-1.74	down
F6WVD1	protein S28	30.4	7.84	-0.58	-1.11	-0.91	1.06	1.15	0.39	2.44	-1.73	down
Q1RMX7	acid synthase	8.4	40.22	-1.26	-0.32	-1.02	0.86	0.82	0.92	2.44	-1.73	down
F6ZRH8	Uncharacterized protein	5.7	101.11	0.96	0.87	0.77	-0.90	-1.34	-0.37	2.43	1.73	up
F1MWM8	receptor C-type 1	10	164.40	-1.03	-1.24	-0.33	0.93	0.70	0.97	2.43	-1.73	down
A7E3W4	Transketolase	3	64.88	-0.30	-1.10	-1.19	0.93	0.88	0.80	2.43	-1.73	down
Q2HJ49	Moesin	16.8	67.97	-1.22	-0.40	-0.99	0.70	1.16	0.74	2.43	-1.73	down
A0A3Q1LT23	Ribosomal_S5_C domain-containing protein	15.2	8.76	-0.56	-0.82	-1.22	1.21	0.48	0.92	2.42	-1.73	down
Q862D8	to S3 ribosomal protein	24.1	16.08	-0.55	-1.31	-0.74	1.21	0.73	0.66	2.42	-1.73	down
A0A0B4J1C4	chain of multimeric IgA and IgM	47.5	17.85	0.46	1.03	1.11	-1.23	-0.53	-0.85	2.41	1.73	up
F6SUZ2	UDP-glucose 4-epimerase	14.7	38.24	-1.18	-0.55	-0.86	1.16	1.02	0.41	2.38	-1.73	down
A0A3Q2H8Y8	Ecto-5'-nucleotidase	3.5	80.66	0.83	0.90	0.86	-0.85	-0.36	-1.38	2.37	1.73	up
F6SX98	aldolase	21.7	39.46	-0.88	-1.00	-0.71	1.27	1.01	0.31	2.36	-1.73	down
K9K4R9	proteasome non-ATPase regulatory subunit 1-like protein (Fragment)	2.3	43.80	-1.12	-0.72	-0.74	0.37	0.92	1.29	2.36	-1.73	down
F6QAQ9	protein	0.4	207.92	-0.86	-1.35	-0.37	1.03	0.71	0.85	2.36	-1.73	down
A0A3Q2IED5	binding protein	0.9	138.99	-0.51	-0.63	-1.45	0.77	0.93	0.89	2.35	-1.73	down
K9K202	division control protein 42-like protein	37.2	21.26	-1.06	-0.78	-0.75	0.40	1.38	0.81	2.34	-1.72	down
F7DQS6	Phosphoglycerate mutase	16.5	28.82	-0.33	-0.92	-1.33	1.02	0.84	0.72	2.31	-1.72	down
F6Q6E8	binding cassette subfamily G member 2	6.9	72.81	0.56	0.92	1.11	-0.47	-1.35	-0.76	2.31	1.72	up
A0A3Q1M0U9	endoplasmic reticulum ATPase	9.5	88.78	-1.23	-0.25	-1.09	0.90	0.89	0.79	2.29	-1.72	down
Q32PD5	ribosomal protein S19	22.1	16.06	-1.27	-0.95	-0.36	1.09	0.92	0.57	2.29	-1.72	down

Q862R3	to ribosomal protein L18a (Fragment)	11.4	18.31	-0.90	-0.33	-1.35	0.96	0.94	0.67	2.29	-1.72	down
A0A140T8A5	dehydrogenase [NADP]	17.1	46.76	-0.73	-1.42	-0.42	0.93	0.96	0.68	2.28	-1.72	down
Q2KJ51	Angiopoietin-related protein 4	2.9	45.55	-0.51	-0.70	-1.37	0.52	1.07	0.98	2.28	-1.72	down
H9GZQ3	domain-containing protein	9.5	11.46	0.32	1.17	1.08	-0.83	-0.59	-1.15	2.26	1.71	up
F1MU79	isomerase	2.2	51.56	-0.74	-0.39	-1.44	0.96	0.90	0.71	2.25	-1.71	down
Q9GKX8	shock protein HSP 90-beta	29.1	83.24	-0.89	-1.31	-0.36	1.13	0.58	0.86	2.23	-1.71	down
F7CGP9	matrix protein 2	3.1	78.66	0.90	0.68	0.99	-0.93	-0.29	-1.35	2.22	1.71	up
F6UME7	factor 1-alpha	39.2	50.14	-0.63	-1.26	-0.68	1.35	0.56	0.66	2.22	-1.71	down
A0A3Q2I8J7	lectin domain-containing protein	8.3	21.39	0.47	1.13	0.96	-1.30	-0.41	-0.86	2.22	1.71	up
F6RI47	Alpha-2-macroglobulin	14.7	164.04	1.15	0.21	1.20	-0.82	-0.91	-0.83	2.20	1.71	up
Q3ZBY8	4,6-dehydratase	8.3	41.86	-0.31	-0.82	-1.43	0.89	0.85	0.81	2.20	-1.71	down
C6L1J5	dehydrogenase	24	36.52	-0.52	-1.50	-0.54	0.90	0.90	0.76	2.20	-1.71	down
F1MUP9	Vesicle amine transport 1	3.7	42.83	-0.29	-0.91	-1.36	0.85	1.05	0.66	2.18	-1.70	down
F6SRP7	cyclase-associated protein	6.3	51.37	-1.10	-0.54	-0.91	1.41	0.48	0.67	2.18	-1.70	down
Q5XWB8	receptor	12.3	37.95	0.90	0.94	0.71	-0.21	-1.20	-1.15	2.17	1.70	up
K9K4B7	shock 70 kDa protein 13-like protein	19.1	46.51	0.78	0.90	0.88	-0.22	-1.01	-1.33	2.17	1.70	up
Q862J2	to phospholipid hydroperoxide glutathione peroxidase	14.8	9.28	-0.21	-1.04	-1.31	0.89	0.81	0.86	2.16	-1.70	down
F7DRP9	domain-containing protein	6.5	23.38	-0.53	-0.95	-1.06	1.21	1.07	0.27	2.15	-1.70	down
E1BB48	N-acetylgalactosaminyltransferase	3.7	58.42	-1.32	-0.51	-0.72	0.60	1.31	0.64	2.15	-1.70	down
A3KMV5	modifier-activating enzyme 1	5.5	117.83	-0.66	-0.95	-0.94	1.45	0.75	0.35	2.15	-1.70	down
A6BMK7	Sialidase-1	5.1	45.43	-0.90	-0.26	-1.39	0.84	1.00	0.71	2.14	-1.70	down
K9KBF9	lipid-transfer protein-like protein	3.8	31.06	-0.68	-0.65	-1.21	0.64	1.42	0.48	2.11	-1.69	down
A0A3Q2LT54	binding protein 2	5.5	62.74	-1.20	-0.18	-1.16	0.76	0.77	1.01	2.09	-1.69	down
F6ZD04	glucan phosphorylase	5	96.41	-1.15	-0.16	-1.22	0.94	0.82	0.76	2.09	-1.69	down
K9K2F9	Peroxiredoxin-1-like protein	24.4	18.42	-0.76	-1.16	-0.60	1.43	0.72	0.38	2.06	-1.69	down
G3N0K3	ribosomal protein S4	7.6	29.48	-0.73	-1.46	-0.33	1.00	0.94	0.59	2.06	-1.68	down
A0A3Q2GVQ5	precursor	10.8	54.31	0.45	0.58	1.50	-0.61	-1.06	-0.85	2.04	1.68	up
B0JYN3	dehydrogenase	24	36.72	-1.02	-0.86	-0.64	1.52	0.54	0.46	2.03	-1.68	down
F6RCA8	Peroxiredoxin-5	17.9	15.64	-0.18	-1.04	-1.30	1.07	0.80	0.64	2.02	-1.68	down
E1BBX5	oxidase 3L1	0.7	148.22	-1.39	-0.93	-0.20	0.76	1.04	0.71	2.01	-1.68	down
F7BL38	domain-containing protein	7.7	71.49	0.69	1.15	0.68	-1.17	-1.16	-0.19	2.01	1.68	up
A0A3Q1MGQ2	Rho GTPase activating protein 18	6	69.96	-1.50	-0.35	-0.66	0.99	0.91	0.62	2.00	-1.67	down
A0A3Q2L483	translation elongation factor 1 delta	4	68.08	-1.20	-0.11	-1.20	0.92	0.83	0.76	2.00	-1.67	down
Q0IIH5	Nucleobindin 2	23.6	49.19	-1.50	-0.25	-0.75	0.86	0.92	0.73	1.99	-1.67	down

F6PNM1	cell derived factor 4	11.2	98.33	0.49	1.45	0.57	-0.54	-1.22	-0.75	1.99	1.67	up
F6Y4I1	uridylyltransferase	31.1	56.55	-0.99	-1.32	-0.19	1.13	0.61	0.77	1.98	-1.67	down
A4URH1	Superoxide dismutase	11.8	10.07	-1.26	-0.85	-0.40	1.36	0.45	0.70	1.98	-1.67	down
F6QUF7	Lipoprotein lipase	21.7	52.72	-1.49	-0.77	-0.24	0.66	0.92	0.92	1.96	-1.67	down
F7CWC8	Amine oxidase	48.7	59.07	0.90	0.94	0.66	-1.16	-1.23	-0.11	1.96	1.67	up
F7CIM1	Stratifin	12.1	27.79	-1.16	-0.11	-1.23	0.88	0.93	0.69	1.96	-1.67	down
F6V2P8	member RAS oncogene family	5.2	23.50	-0.52	-0.80	-1.18	0.32	1.44	0.73	1.93	-1.66	down
F7A5X7	serine/threonine protein kinase	0.5	191.13	-1.32	-0.28	-0.89	1.20	0.88	0.40	1.91	-1.66	down
O77811	Lactotransferrin	55.7	75.99	-1.43	-0.40	-0.65	1.12	0.97	0.39	1.90	-1.66	down
F6WC66	Dihydropyrimidinase like 3	14.6	61.94	-0.45	-0.50	-1.53	1.13	0.66	0.69	1.90	-1.66	down
F6YV53	Actinin alpha 4	10.9	99.85	-0.13	-1.44	-0.90	0.72	0.94	0.82	1.89	-1.65	down
Q862S8	to ribosomal protein S18	15.2	14.76	-1.12	-1.28	-0.08	1.00	0.75	0.74	1.89	-1.65	down
F7CAB8	1, zinc-binding	12.8	35.48	0.46	1.07	0.96	-0.57	-1.50	-0.41	1.89	1.65	up
E1BMG9	dehydrogenase	6	98.75	-1.10	-0.11	-1.27	1.11	0.67	0.70	1.88	-1.65	down
F7DG10	H+ transporting accessory protein 1	3	51.59	-1.51	-0.41	-0.56	1.07	0.93	0.47	1.88	-1.65	down
K9K252	Calmodulin-like protein	15.8	15.64	-1.58	-0.45	-0.44	0.84	0.97	0.67	1.88	-1.65	down
Q3MHP2	protein Rab-11B	31.2	24.49	-0.05	-1.18	-1.23	0.99	0.75	0.73	1.85	-1.65	down
F6ZUJ2	Endoplasmin-like protein	11.8	92.42	-0.99	-0.94	-0.53	1.18	1.20	0.08	1.84	-1.64	down
H9GZQ9	Uncharacterized protein	32.4	48.11	0.90	0.90	0.67	-1.23	-0.03	-1.20	1.82	1.64	up
A0A3Q1MFR4	Apolipoprotein B	0.9	514.08	-0.46	-1.56	-0.43	0.50	1.00	0.95	1.80	-1.64	down
A0A140T8A7	pseudouridine synthase D4	1.9	42.06	-0.72	-0.19	-1.54	0.98	0.61	0.85	1.79	-1.63	down
B7UBT6	motif chemokine	25.7	11.74	-1.50	-0.08	-0.87	0.80	0.78	0.87	1.78	-1.63	down
A2VDX0	TWF2 protein	3.7	39.56	-0.28	-0.56	-1.60	0.98	0.81	0.65	1.77	-1.63	down
A0A3Q2L3I3	Clusterin	21.5	50.88	-1.51	-0.85	-0.08	0.79	0.83	0.82	1.77	-1.63	down
K9K395	pyrophosphatase-like protein	14.7	29.98	-0.74	-0.34	-1.36	1.11	1.10	0.23	1.76	-1.63	down
P00559	Phosphoglycerate kinase 1	29.7	44.60	-0.54	-0.44	-1.45	1.34	0.58	0.52	1.76	-1.63	down
F6X1I8	Hemopexin	18.8	51.36	0.80	0.73	0.91	-0.01	-1.38	-1.05	1.75	1.62	up
P00570	kinase isoenzyme 1	9.8	21.66	-0.34	-0.73	-1.36	0.54	1.40	0.49	1.75	-1.62	down
Q0QEV1	protein L18	23.6	17.82	-1.32	-0.08	-1.03	1.20	0.67	0.56	1.73	-1.62	down
A0A3Q2I2R2	subunit alpha type	12.3	11.66	-0.72	-1.55	-0.16	0.91	0.57	0.95	1.73	-1.62	down
F6PH25	phosphodiesterase 1	4	91.06	1.02	0.74	0.67	-0.02	-1.40	-1.01	1.73	1.62	up
K9KCM0	Fructose-bisphosphate aldolase	8.6	28.93	-1.30	-0.10	-1.02	1.24	0.55	0.63	1.71	-1.61	down
F6TD06	ADP ribosylation factor 3	39.2	20.60	-0.70	-1.59	-0.14	0.70	0.84	0.88	1.71	-1.61	down
F6VKZ3	domain-containing protein	11.6	14.93	-1.29	0.01	-1.15	0.69	1.02	0.71	1.71	-1.61	down
F6WFS0	Ephrin A1	6.6	35.87	1.09	0.92	0.41	-1.55	-0.33	-0.55	1.71	1.61	up
P17248	ligase, cytoplasmic	6.7	53.81	-0.16	-0.97	-1.28	1.32	0.66	0.44	1.71	-1.61	down
F6RRV1	Fetuin B	10.2	39.78	0.75	0.65	1.02	-1.38	0.00	-1.04	1.71	1.61	up
K9K259	multivesicular body protein 5-like protein	6.1	18.95	0.92	0.78	0.72	-0.48	-1.65	-0.29	1.70	1.61	up
A0A3Q2GTT0	domain-containing protein	21.7	11.64	-0.37	-1.59	-0.45	1.03	0.45	0.94	1.70	-1.61	down

A0A3Q2H4H0	specific peptidase 7	1.2	117.54	-0.96	-0.81	-0.64	1.22	1.23	-0.04	1.69	-1.61	down
F7BXD8	molecule like	2.9	37.99	0.96	0.83	0.61	-1.65	-0.36	-0.40	1.69	1.61	up
F7D8I6	Xanthine dehydrogenase	32.2	146.77	-1.38	-0.84	-0.19	0.30	1.18	0.93	1.68	-1.61	down
F6XA25	Perilipin 3	8.1	57.59	-0.34	-1.61	-0.46	1.00	0.49	0.91	1.68	-1.61	down
A0A3Q1M119	protein epsilon	29.2	27.42	-1.08	-1.37	0.05	0.89	0.79	0.73	1.68	-1.60	down
F1MBV2	Ribose-phosphate diphosphokinase	5.2	35.66	-0.73	-0.17	-1.51	1.18	0.53	0.69	1.67	-1.60	down
H9GZR2	Uncharacterized protein	22.9	47.16	0.90	0.76	0.73	0.07	-1.36	-1.10	1.65	1.60	up
F6TIR2	Lipocalin 2	36	33.91	0.46	0.28	1.65	-1.01	-0.67	-0.71	1.64	1.59	up
Q5E9A3	Poly(rC)-binding protein 1	7.6	37.50	-1.33	-1.06	0.00	0.92	1.04	0.43	1.63	-1.59	down
F7DMU4	necrosis factor receptor superfamily member 11B	3.5	46.24	-0.34	-1.38	-0.66	0.34	0.62	1.42	1.62	-1.59	down
A0A3Q2L4D4	subunit beta 8	5.8	26.14	-1.07	0.03	-1.34	1.10	0.73	0.56	1.62	-1.59	down
F6XWM5	Haptoglobin	38.3	38.47	0.85	0.51	1.03	-0.02	-1.48	-0.88	1.62	1.59	up
B5BV06	Alpha-1-antitrypsin	25.4	46.82	0.62	0.97	0.79	-1.42	-1.01	0.05	1.61	1.59	up
A0A3Q1LG13	Uncharacterized protein	0.3	423.52	-0.77	-1.33	-0.28	0.47	1.46	0.45	1.61	-1.59	down
F1MQG4	finger DBF-type containing 2	0.8	274.06	-0.66	-0.92	-0.80	1.29	1.18	-0.09	1.61	-1.59	down
F1MXZ0	N-acetylglucosamine-6-sulfatase	3.8	62.80	-1.49	-0.02	-0.87	0.55	0.74	1.09	1.60	-1.59	down
A0A0A1E482	lambda light chain variable region	29	23.34	-0.68	-0.88	-0.81	1.17	1.31	-0.10	1.60	-1.58	down
A0A3Q1M4K6	Fibronectin	7.9	237.57	0.06	-1.26	-1.17	0.50	0.80	1.07	1.59	-1.58	down
Q862E2	to acidic ribosomal phosphoprotein PO	12.1	17.03	-0.82	-0.80	-0.75	1.56	0.82	-0.01	1.59	-1.58	down
F6PQ46	Ceruloplasmin	16.7	121.12	1.04	0.49	0.84	-0.30	-1.64	-0.42	1.59	1.58	up
Q3SX32	Perilipin	5.5	47.60	0.09	-0.96	-1.48	0.64	0.84	0.88	1.55	-1.57	down
Q6GVI4	MHC class II antigen	12.9	10.65	-1.02	-0.87	-0.46	-0.08	1.12	1.31	1.54	-1.57	down
A0A3Q2HFS3	containing TCP1 subunit 2	5.6	57.47	-0.56	-1.03	-0.75	1.54	0.83	-0.03	1.53	-1.56	down
F6UPZ6	RAB6A	5.3	23.52	-1.26	-1.24	0.17	0.79	0.87	0.68	1.51	-1.56	down
K9K313	ribosomal protein L4-like protein	5.3	42.18	-0.59	-0.78	-0.96	-0.14	1.37	1.11	1.50	-1.55	down
F7DTB6	subunit beta 1	1	97.23	-1.60	-0.72	-0.01	1.04	0.66	0.63	1.50	-1.55	down
F6ZAZ2	Dicarbonyl and L-xylulose reductase	4.1	25.74	-0.72	-0.11	-1.49	1.04	0.24	1.05	1.50	-1.55	down
A0A3Q2HTF1	associated protein 23	7.2	11.09	-0.66	-0.79	-0.88	0.65	1.66	0.02	1.49	-1.55	down
F7BMS8	transferase subunit gamma	4.4	37.79	0.52	1.04	0.75	0.16	-1.25	-1.22	1.47	1.54	up
Q861K5	class I antigen	9.6	30.26	0.38	0.29	1.64	-0.85	-1.11	-0.36	1.46	1.54	up
F7BFJ1	Prothrombin	9.9	69.64	1.26	0.51	0.54	-0.48	-1.60	-0.23	1.46	1.54	up
Q60FB3	protein P-B	37.5	5.68	0.86	0.79	0.65	-1.61	-0.78	0.08	1.45	1.54	up
F7CZ92	alpha 1, skeletal muscle	27.6	42.05	-0.47	-1.72	-0.11	0.91	0.78	0.61	1.43	-1.53	down
P48644	dehydrogenase 1	7.2	54.81	-0.98	0.15	-1.45	0.81	1.01	0.46	1.42	-1.53	down
K9K2E5	disulfide-isomerase	18.2	48.00	0.92	0.36	1.01	0.11	-1.45	-0.94	1.42	1.53	up

F6X8Q2	Phosphoglucomutase 1	8.9	61.55	-1.34	-1.16	0.21	0.98	0.68	0.62	1.41	-1.52	down
F6V6F7	Exo-alpha-sialidase	6.5	45.44	-1.51	-0.94	0.17	0.99	0.60	0.68	1.40	-1.52	down
F7CYG2	3-monooxygenase/tryptophan 5-monooxygenase activation protein eta	13.8	28.21	-1.68	-0.63	0.03	0.78	0.92	0.59	1.40	-1.52	down
Q1RMT9	Phosphoinositide-3-kinase-interacting protein 1	5.7	27.77	-1.56	0.12	-0.84	0.51	0.98	0.79	1.40	-1.52	down
E1BAN2	p60 ATPase-containing subunit A-like 2	2.5	53.91	-0.61	-1.29	-0.38	1.23	1.14	-0.10	1.39	-1.52	down
A5D7E8	disulfide-isomerase	11.9	56.93	-0.60	0.02	-1.70	0.60	0.85	0.83	1.39	-1.52	down
P45478	thioesterase 1	4.9	34.14	-0.10	-1.24	-0.92	0.24	1.50	0.53	1.38	-1.51	down
F7BM31	family D member 1	7.1	56.69	1.64	0.60	0.02	-0.35	-0.92	-0.99	1.36	1.51	up
A0A3Q2ICN7	Uncharacterized protein	32.2	70.90	0.07	-1.19	-1.13	0.13	1.17	0.96	1.36	-1.50	down
F7CQ86	domain-containing protein	4.5	23.16	-0.19	-0.51	-1.55	1.36	0.28	0.61	1.35	-1.50	down
K9KFE3	ribosomal protein L6	10.6	33.95	-0.98	-0.93	-0.34	1.71	0.19	0.35	1.34	-1.50	down
Q5E9E6	ribosomal protein L10a	6	24.83	-0.65	-0.05	-1.55	1.22	0.80	0.23	1.34	-1.50	down
F6ZFH9	3-monooxygenase/tryptophan 5-monooxygenase activation protein gamma	16.2	28.30	-0.93	0.20	-1.52	0.98	0.51	0.75	1.34	-1.50	down
F6ZTR4	Tropomyosin 3	15.6	26.85	-1.24	0.20	-1.20	0.66	1.17	0.41	1.34	-1.50	down
F6RA08	Stomatin	24.3	31.24	-1.03	-0.38	-0.84	1.48	-0.17	0.93	1.34	-1.50	down
A0A3Q2KZE9	motif containing GTPase activating protein 1	2.7	182.80	-0.12	-1.55	-0.57	1.00	1.11	0.12	1.33	-1.49	down
Q1JPD9	protein-coupled receptor, family C, group 5, member B	6.2	43.07	0.18	-1.63	-0.79	0.71	0.80	0.74	1.33	-1.49	down
P80025	Lactoperoxidase	2.8	80.64	-0.82	-0.45	-0.96	1.65	0.68	-0.10	1.32	-1.49	down

1.Detailed information of the differentially expressed proteins (DM vs HM)												
**UniProt Accession**	**Protein Name**	**Coverage**	**Mol/Weight**	**LFQ intensity**						**-Log(p-value)**	**Log2(fold change)**	**Change**
				**DM-1**	**DM-2**	**DM-3**	**HM-1**	**HM-2**	**HM-3**			
F6Z2L5	Apolipoprotein E	54.1	30.33	0.94	0.89	0.90	-0.94	-0.93	-0.87	6.66	1.83	up
H9GZT5	Uncharacterized protein	33.2	43.03	0.90	0.89	0.94	-0.86	-0.93	-0.95	6.22	1.82	up
A0A3Q2LNC9	intracellular cholesterol transporter 2	31.4	16.50	0.89	0.96	0.89	-0.85	-0.96	-0.92	5.95	1.82	up
F6VUW2	Cathepsin S	19.6	37.32	0.85	0.95	0.94	-0.95	-0.93	-0.86	5.82	1.82	up
P23381	ligase, cytoplasmic	3.6	53.17	-0.84	-0.97	-0.92	0.87	0.91	0.95	5.75	-1.82	down
F7C7V8	Semaphorin 7A	25.6	74.53	0.91	0.96	0.87	-0.88	-0.99	-0.86	5.58	1.82	up
F7CAB8	zinc-binding	15.7	35.48	-0.91	-0.87	-0.95	0.99	0.85	0.90	5.55	-1.82	down
P08334	Alpha-lactalbumin A	91.1	14.22	0.85	0.95	0.93	-0.84	-0.97	-0.92	5.48	1.82	up
A0A3Q2HN20	albumin	77.9	66.74	0.92	0.95	0.86	-0.82	-0.96	-0.96	5.38	1.82	up
Q99541	Perilipin-2	12.4	48.08	-0.81	-0.97	-0.96	0.91	0.89	0.93	5.34	-1.82	down
F7BL38	domain-containing protein	7.7	71.49	0.91	0.93	0.89	-0.88	-0.83	-1.03	5.12	1.82	up
A6NC98	domain-containing protein 88B	0.5	164.81	-1.01	-0.83	-0.88	0.97	0.88	0.88	5.11	-1.82	down
F6X1I8	Hemopexin	16.9	51.36	0.85	0.87	1.01	-0.98	-0.92	-0.83	5.00	1.82	up
F7B0S3	Milk fat globule EGF and factor V/VIII domain containing	40	54.87	0.86	0.85	1.02	-0.84	-0.97	-0.91	4.94	1.82	up
Q6X9W5	amyloid A protein	41.7	13.28	0.97	0.87	0.88	-1.03	-0.80	-0.89	4.81	1.82	up
A0A3Q2I2H1	domain-containing protein	38	10.65	0.91	0.94	0.88	-0.78	-0.93	-1.03	4.77	1.82	up
P86273	Beta-casein	18.6	25.53	0.87	1.00	0.86	-1.01	-0.81	-0.91	4.76	1.82	up
Q08431	Lactadherin	5.7	43.10	-0.99	-0.81	-0.94	1.01	0.82	0.90	4.69	-1.82	down
A0A0A1E417	lambda light chain variable region	24.9	23.43	0.87	0.93	0.93	-0.76	-1.03	-0.94	4.61	1.82	up
F6W3K7	Symplekin	1.1	140.61	-1.00	-0.75	-0.98	0.91	0.86	0.96	4.59	-1.82	down
K9K2R3	Annexin	10.9	29.09	-0.89	-1.06	-0.78	0.92	0.92	0.88	4.59	-1.82	down
A0A3Q2KY85		16.7	32.20	0.92	0.80	1.01	-0.93	-0.81	-0.99	4.58	1.82	up
Q95M34	gamma 1 heavy chain constant region	45.4	37.44	0.90	0.96	0.87	-1.06	-0.79	-0.87	4.56	1.82	up
P11376	C, milk isozyme	74.4	14.65	0.91	0.92	0.90	-0.94	-0.75	-1.03	4.55	1.82	up

A0A3Q2H8T4	protein	26.4	183.88	1.04	0.78	0.91	-0.97	-0.82	-0.93	4.49	1.82	up
F6YL06	protein subunit beta 1	10.6	37.35	-1.08	-0.81	-0.84	0.91	0.89	0.93	4.47	-1.82	down
F6VF11	Chordin like 2	22.6	46.35	0.94	0.88	0.91	-0.76	-0.91	-1.06	4.47	1.82	up
Q9Y4Z0	snRNA-associated Sm-like protein LSm4	5	15.35	-0.91	-0.77	-1.04	0.96	0.82	0.94	4.45	-1.82	down
A0A3Q2KT61	4,5-bisphosphate phosphodiesterase gamma	1.1	144.43	0.88	0.93	0.91	-0.74	-1.04	-0.95	4.44	1.82	up
A0A3Q2H4E3	carrier family 34 member 2	7.5	79.24	0.80	0.93	1.00	-0.84	-0.84	-1.05	4.43	1.82	up
Q99536	vesicle membrane protein VAT-1 homolog	10.9	41.92	-1.06	-0.93	-0.74	0.92	0.89	0.92	4.39	-1.82	down
F6W1N4	immunoglobulin receptor	33.2	83.00	0.89	0.95	0.88	-0.72	-0.98	-1.02	4.35	1.82	up
A0A3Q2HNJ8	protein	19.4	139.94	-0.94	-0.74	-1.05	0.85	0.93	0.95	4.34	-1.82	down
A0A3Q2GVQ5	Granulin precursor	10.8	54.31	0.86	0.97	0.90	-1.00	-0.73	-1.00	4.33	1.82	up
F7DXM5	domain-containing protein	10.9	46.76	0.96	0.91	0.86	-1.09	-0.85	-0.78	4.29	1.82	up
A0A3Q2H536	antigen	6	66.30	0.84	0.97	0.90	-0.72	-0.99	-1.01	4.26	1.81	up
A0A0A1E691	lambda light chain variable region	28.7	23.04	0.90	0.96	0.86	-1.07	-0.73	-0.93	4.21	1.81	up
O97973	Transferrin	21.3	6.88	0.83	0.94	0.95	-0.99	-0.72	-1.02	4.20	1.81	up
F6ZI35	Histidine rich glycoprotein	5.4	57.93	0.87	1.02	0.83	-1.03	-0.73	-0.96	4.15	1.81	up
P17050	Alpha-N-acetylgalactosaminidase	8.3	46.56	0.93	0.94	0.86	-1.04	-0.69	-0.99	4.10	1.81	up
P86272	Alpha-S1-casein	30.2	24.41	0.84	1.09	0.79	-1.01	-0.90	-0.80	4.09	1.81	up
P47989	dehydrogenase/oxidase	18.3	146.42	-0.86	-0.74	-1.12	0.91	0.92	0.89	4.09	-1.81	down
Q02818	Nucleobindin-1	36.9	53.88	-1.06	-0.96	-0.70	0.87	0.90	0.94	4.09	-1.81	down
H9GZQ9	Uncharacterized protein	34.7	48.11	0.91	0.90	0.91	-1.02	-1.02	-0.68	4.09	1.81	up
F7DGX8	Cartilage acidic protein 1	3.6	66.78	-0.73	-1.05	-0.93	0.80	1.01	0.91	4.07	-1.81	down
P15311	Ezrin	7.5	69.41	-0.70	-0.98	-1.04	0.98	0.88	0.86	4.06	-1.81	down
F6YYP6	domain-containing protein	41.4	11.19	1.05	0.82	0.84	-0.84	-0.81	-1.08	4.06	1.81	up
A0A3Q2KRR2	CD55 molecule	13.9	92.46	0.89	0.94	0.88	-0.69	-1.07	-0.96	4.05	1.81	up
P02751	Fibronectin	4.5	262.62	-0.87	-0.89	-0.96	1.10	0.90	0.72	4.02	-1.81	down
B5BV07	Alpha-1-antitrypsin	20.7	46.91	0.76	1.01	0.95	-0.86	-0.78	-1.08	3.99	1.81	up
F6VB94	growth factor beta induced	28.6	74.31	0.84	0.90	0.97	-0.71	-0.90	-1.11	3.96	1.81	up
P62491	protein Rab-11A	19.4	24.39	-1.00	-1.04	-0.67	0.93	0.91	0.87	3.93	-1.81	down

A0A0B4J1C4	chain of multimeric IgA and IgM	47.5	17.85	0.68	1.00	1.04	-0.84	-0.98	-0.90	3.93	1.81	up
B5BV04	Alpha-1-antitrypsin	21.1	46.86	0.95	0.89	0.88	-0.67	-0.99	-1.06	3.92	1.81	up
C0HJR4	Lactadherin	100	2.60	0.82	0.86	1.03	-1.08	-0.92	-0.72	3.92	1.81	up
B1PLB8	Kappa-casein	43.9	14.90	0.86	1.04	0.81	-1.11	-0.81	-0.79	3.88	1.81	up
Q99943	acyltransferase alpha	10.6	31.72	-0.73	-0.91	-1.07	0.90	0.77	1.04	3.88	-1.81	down
F6X6A6	cis-trans isomerase	31.7	17.89	-1.11	-0.90	-0.71	1.00	0.89	0.82	3.87	-1.81	down
K9KDJ7	transport protein 81-like protein-like protein	1.5	53.09	-1.05	-0.96	-0.70	0.75	0.95	1.01	3.78	-1.81	down
A0A3Q2L2R4	domain-containing protein	4.9	20.72	0.95	0.93	0.83	-0.70	-0.87	-1.14	3.78	1.81	up
P08896	Alpha-lactalbumin B/C	42.3	14.25	0.94	0.89	0.88	-1.09	-0.65	-0.98	3.75	1.81	up
P02810	acidic proline-rich phosphoprotein 1/2	10.2	17.02	0.97	1.06	0.68	-0.92	-0.77	-1.02	3.73	1.81	up
F6VBP9	Apolipoprotein E	15.2	34.97	0.80	1.08	0.83	-0.79	-1.11	-0.81	3.72	1.81	up
Q9NS61	channel-interacting protein 2	7	30.91	0.68	0.87	1.15	-0.95	-0.86	-0.91	3.71	1.80	up
F7BPX8	Cathepsin L1	36.2	37.40	0.85	1.01	0.85	-1.03	-0.64	-1.04	3.67	1.80	up
A0A3Q2HNI5	II alpha subunit	8.2	101.16	-1.16	-0.82	-0.72	0.88	0.83	1.00	3.65	-1.80	down
B7VGF9	Alpha-S2-casein	69.9	27.70	0.92	1.04	0.74	-0.73	-0.87	-1.11	3.65	1.80	up
F6ZNX3	type XIV alpha 1 chain	0.7	192.87	-0.81	-1.10	-0.80	0.91	0.72	1.08	3.64	-1.80	down
F7BM31	family D member 1	7.1	56.69	0.98	0.98	0.74	-1.03	-1.01	-0.66	3.64	1.80	up
F6ZRH8	Uncharacterized protein	5.7	101.11	1.03	0.94	0.74	-1.12	-0.72	-0.86	3.61	1.80	up
P55259	secretory granule membrane major glycoprotein GP2	3.5	59.48	-0.84	-1.16	-0.71	0.96	0.78	0.96	3.61	-1.80	down
F5CEP2	fat globule-EGF factor 8 splice variant	52.1	43.31	0.84	0.79	1.07	-1.05	-0.99	-0.66	3.58	1.80	up
P05413	acid-binding protein, heart	35.3	14.86	-1.09	-0.95	-0.66	0.97	0.75	0.98	3.58	-1.80	down
F7BXD8	CD5 molecule like	7.5	37.99	1.17	0.88	0.65	-0.91	-0.90	-0.89	3.56	1.80	up
F6RI47	Alpha-2-macroglobulin	16.4	164.04	0.90	0.84	0.96	-1.13	-0.95	-0.63	3.55	1.80	up
F6U904	Glycoprotein IIIb	11.3	48.34	-0.81	-0.93	-0.96	1.12	0.63	0.95	3.54	-1.80	down
A0A3Q2HSB3	Lactoperoxidase	10	78.11	-0.72	-1.20	-0.78	0.90	0.90	0.90	3.53	-1.80	down
A0A3Q2ID55	carrier family 4 member 9	1	95.76	0.85	0.89	0.96	-1.17	-0.65	-0.89	3.52	1.80	up
A0A3Q2I0A2	Fibroblast growth factor-binding protein 1	20.8	24.30	0.92	0.84	0.94	-1.15	-0.63	-0.92	3.52	1.80	up
A0A3Q2I5T2	domain-containing protein	10.6	15.20	0.95	0.67	1.08	-0.74	-1.07	-0.89	3.51	1.80	up

Q5XWB8	Monocyte differentiation antigen CD14	12.3	37.95	0.83	0.90	0.97	-1.09	-0.61	-1.00	3.50	1.80	up
F6RM73	Apolipoprotein A-II	18.5	17.40	0.95	0.87	0.88	-0.59	-1.04	-1.06	3.50	1.80	up
F7CGP9	matrix protein 2	4.3	78.66	0.95	0.70	1.04	-1.01	-0.65	-1.03	3.45	1.80	up
A0A3Q2L3I3	Clusterin	19.2	50.88	0.87	0.74	1.09	-1.05	-0.65	-0.99	3.43	1.80	up
A0A3Q2HWQ6	C3-beta-c	31.5	171.14	0.95	0.77	0.97	-0.60	-1.00	-1.09	3.42	1.80	up
A0A3Q2H027	endoplasmic reticulum lectin	11.7	69.38	-0.78	-1.13	-0.79	0.82	0.75	1.13	3.40	-1.80	down
A0A3Q2I560	cotransporter	12.7	86.31	0.84	0.85	1.00	-1.21	-0.69	-0.80	3.39	1.80	up
P01833	immunoglobulin receptor	1	83.28	-0.58	-1.03	-1.08	0.97	0.90	0.82	3.38	-1.80	down
H9GZU8	Uncharacterized protein	39.1	48.92	0.92	0.84	0.93	-0.57	-1.04	-1.08	3.37	1.79	up
A0A0A1E6I9	lambda light chain variable region	22	23.33	-0.91	-0.61	-1.17	0.99	0.86	0.85	3.36	-1.79	down
H9GZR2	Uncharacterized protein	18.2	47.16	0.96	0.90	0.82	-0.64	-0.84	-1.21	3.33	1.79	up
F7CSL8	domain-containing protein	14.5	46.87	0.91	0.82	0.95	-0.56	-1.05	-1.08	3.32	1.79	up
P48723	shock 70 kDa protein 13	10	51.93	-0.89	-1.14	-0.66	1.02	0.70	0.97	3.32	-1.79	down
F7B3U1	protein-coupled receptor class C group 5 member B	6.2	43.04	-1.10	-0.99	-0.60	0.79	0.84	1.05	3.31	-1.79	down
F6QXW2	binding protein 1	21.9	22.30	-0.98	-1.14	-0.57	0.85	0.93	0.91	3.31	-1.79	down
A5YBL8	cis-trans isomerase	31.5	23.81	0.98	0.68	1.03	-0.74	-0.79	-1.16	3.30	1.79	up
C1L3G3	s2 casein B	20	18.36	0.80	1.21	0.68	-1.02	-0.77	-0.90	3.30	1.79	up
P28546	Alpha-lactalbumin	91.1	14.22	0.91	0.92	0.86	-0.56	-1.00	-1.13	3.30	1.79	up
F6UL68	Transthyretin	19.4	15.50	0.87	1.08	0.74	-1.18	-0.70	-0.81	3.29	1.79	up
K9KBC2	protein epsilon-like protein	15.5	24.24	-1.22	-0.68	-0.78	0.82	0.87	1.00	3.29	-1.79	down
A0A3Q2HM99	WAP four-disulfide core domain protein 2	13.5	16.39	1.06	0.58	1.04	-0.73	-1.00	-0.96	3.27	1.79	up
F6UZH0	Alpha-1-microglobulin	8.2	39.01	0.95	0.86	0.88	-0.62	-0.84	-1.22	3.26	1.79	up
F6T7K7	Transcobalamin 1	23.8	48.27	0.80	0.89	0.99	-1.22	-0.64	-0.83	3.25	1.79	up
P11375	Lysozyme C	62.8	14.69	0.86	0.93	0.89	-0.54	-1.03	-1.11	3.24	1.79	up
F6PQ46	Ceruloplasmin	16.1	121.12	1.05	0.73	0.90	-1.01	-0.58	-1.09	3.22	1.79	up
A0A3Q2H6M5	like 2	6.2	81.23	-1.26	-0.70	-0.71	0.95	0.81	0.92	3.15	-1.79	down
P34096	Ribonuclease 4	16.3	16.84	-0.62	-1.23	-0.83	0.89	0.78	1.01	3.14	-1.79	down
A0A3Q2GT28	Neural proliferation	3.4	34.80	0.83	0.83	1.01	-1.22	-0.87	-0.59	3.13	1.78	up
P62942	cis-trans isomerase FKBP1A	25	11.95	-0.95	-0.58	-1.15	0.82	0.78	1.08	3.12	-1.78	down

O75874	dehydrogenase [NADP] cytoplasmic	23.2	46.66	-0.61	-1.26	-0.81	0.95	0.87	0.85	3.10	-1.78	down
I3RM62	2-phospho-D-glycerate hydro-lyase	16.8	47.14	-0.96	-0.66	-1.06	0.74	1.20	0.73	3.09	-1.78	down
F7C0D9	Kininogen 1	9.1	47.96	1.11	0.85	0.71	-1.03	-0.58	-1.06	3.09	1.78	up
H9GZQ3	domain-containing protein	9.5	11.46	0.79	0.99	0.89	-0.61	-0.81	-1.25	3.08	1.78	up
F7BXQ8	Histone H2A	35.2	13.55	-1.02	-0.84	-0.81	0.82	1.25	0.60	3.05	-1.78	down
A0A3Q2HIU8	fragment of IgA and IgM receptor	2.2	53.54	0.94	0.61	1.13	-1.14	-0.82	-0.71	3.05	1.78	up
Q8WXI7	Mucin-16	0.3	1519.20	-1.00	-1.16	-0.51	1.00	0.87	0.80	3.03	-1.78	down
F7B821	disulfide-isomerase	18.6	57.24	0.50	0.99	1.18	-0.85	-0.87	-0.95	3.01	1.78	up
A0A3Q2H149	domain-containing protein	5.3	23.13	0.89	0.65	1.13	-1.11	-0.96	-0.59	2.99	1.78	up
F6S3Y7	S100	11.4	8.96	0.67	0.97	1.03	-0.79	-1.23	-0.64	2.99	1.78	up
F6TN81	Lipoprotein lipase	3.4	56.56	-0.93	-0.72	-1.02	0.51	1.10	1.05	2.98	-1.78	down
A0A3Q2L6J7	Cathepsin X	11.4	33.42	-1.12	-1.06	-0.48	0.97	0.81	0.89	2.95	-1.78	down
F6YZK3	domain-containing protein	8.5	11.71	0.89	1.08	0.70	-0.89	-1.20	-0.57	2.95	1.78	up
F6PKE1	Inhibitor of carbonic anhydrase	11.8	80.15	-1.14	-0.65	-0.87	1.21	0.75	0.70	2.93	-1.77	down
P01024	Complement C3	6.4	187.15	0.93	0.69	1.03	-1.00	-1.15	-0.50	2.89	1.77	up
F7DI80	Folate receptor alpha	6.2	18.89	-0.96	-1.22	-0.47	0.84	0.84	0.97	2.85	-1.77	down
A0A3Q2H6L1	cofactor protein	7.2	41.35	0.93	0.92	0.81	-0.52	-0.85	-1.29	2.84	1.77	up
F6RZ27	Apolipoprotein E	10.8	43.25	0.72	0.99	0.95	-0.74	-0.61	-1.30	2.83	1.77	up
F6Y426	Nucleobindin 2	17.6	50.22	1.05	0.85	0.75	-1.26	-0.53	-0.86	2.82	1.77	up
F7BFJ1	Prothrombin	12.3	69.64	0.96	0.91	0.78	-1.18	-1.03	-0.44	2.81	1.77	up
P02774	D-binding protein	13.5	52.92	1.27	0.77	0.60	-0.71	-1.10	-0.84	2.80	1.77	up
A0A3Q2H8Y8	Ecto-5'-nucleotidase	5.8	80.66	0.81	0.92	0.92	-0.54	-1.32	-0.79	2.79	1.77	up
F6T0P6	vitamin D binding protein	29.7	52.70	0.86	1.11	0.68	-0.93	-0.52	-1.20	2.79	1.77	up
P49327	acid synthase	1.5	273.42	-0.67	-1.34	-0.63	0.96	0.91	0.78	2.77	-1.76	down
F6WR95	oxidase	9.3	82.98	-1.16	-1.07	-0.41	0.90	0.84	0.91	2.76	-1.76	down
A0A3Q2GXY5	H+ transporting accessory protein 2	2.3	38.84	-0.92	-1.22	-0.50	0.98	0.65	1.01	2.76	-1.76	down
F6YNH6		38.8	37.72	0.84	1.00	0.80	-1.20	-0.43	-1.02	2.74	1.76	up
A0A3Q2KP77	Defensin beta 1	11.8	7.38	1.19	0.75	0.70	-1.09	-0.51	-1.03	2.72	1.76	up
F6RRV1	Fetuin B	10.2	39.78	0.81	0.65	1.18	-1.14	-0.99	-0.52	2.71	1.76	up

Q9H1C7	and transmembrane domain-containing protein 1	10.3	10.63	-0.91	-1.24	-0.49	0.99	0.65	0.99	2.69	-1.76	down
A0A3Q2HTG2	alpha chain	6	77.40	0.75	0.55	1.33	-0.71	-1.00	-0.93	2.67	1.76	up
A0A3Q2KN19	Nucleobindin-1	47.3	53.26	-1.13	-0.97	-0.54	1.13	0.53	0.97	2.66	-1.76	down
F6Y0D9	Cadherin-1	2.2	99.55	1.16	0.79	0.68	-1.16	-0.48	-1.00	2.66	1.76	up
P37837	Transaldolase	2.4	37.54	-1.11	-0.72	-0.80	0.83	1.29	0.51	2.62	-1.75	down
P01860	heavy constant gamma 3	15.1	41.29	1.16	0.87	0.60	-0.51	-1.20	-0.91	2.61	1.75	up
P00559	Phosphoglycerate kinase 1	16.3	44.60	-1.01	-0.50	-1.12	0.73	1.22	0.68	2.61	-1.75	down
Q865P6	Beta-defensin 1	17.2	6.88	1.23	0.63	0.77	-0.72	-0.65	-1.25	2.58	1.75	up
P02758	Beta-lactoglobulin-1	62.2	20.34	0.90	0.88	0.85	-0.40	-0.92	-1.31	2.57	1.75	up
F7DJS6	1-epimerase	8.5	37.67	-0.96	-1.28	-0.38	0.96	0.89	0.77	2.55	-1.75	down
F7C603	phosphodiesterase acid like 3B	4	60.54	0.83	1.22	0.56	-0.82	-0.56	-1.23	2.50	1.74	up
F6UME7	factor 1-alpha	22.7	50.14	-0.84	-1.34	-0.44	0.76	1.05	0.79	2.49	-1.74	down
F6Z4J4	GDP dissociation inhibitor	28.3	50.42	-1.03	-1.15	-0.43	1.05	1.02	0.54	2.49	-1.74	down
A0A3Q2I427	domain-containing protein	12.4	38.30	0.69	0.54	1.38	-1.02	-0.68	-0.91	2.48	1.74	up
P35747	albumin	72	68.60	0.90	0.94	0.77	-0.55	-0.64	-1.41	2.47	1.74	up
A0A3Q2HEN1	inhibitor 3	8.8	22.08	-0.34	-1.30	-0.97	0.92	0.81	0.88	2.44	-1.74	down
F6WG98	Transporter	1.7	71.93	0.75	0.75	1.09	-0.77	-0.46	-1.37	2.41	1.73	up
A0A3Q2ICN7	Uncharacterized protein	31.1	70.90	-1.37	-0.78	-0.45	0.72	1.06	0.82	2.41	-1.73	down
F6PNM1	cell derived factor 4	12.4	98.33	-1.43	-0.70	-0.47	0.92	0.83	0.85	2.41	-1.73	down
F7C0Y4	amyloid A protein	43.8	14.34	1.09	0.71	0.79	-0.77	-0.43	-1.38	2.34	1.72	up
F6WQ61	protein subunit beta 2	7.1	37.33	-0.57	-1.26	-0.76	1.28	0.81	0.50	2.31	-1.72	down
P01023	Alpha-2-macroglobulin	2.2	163.29	0.99	0.56	1.04	-0.42	-0.83	-1.34	2.31	1.72	up
P07900	shock protein HSP 90-alpha	9.2	84.66	-0.80	-1.14	-0.64	0.78	1.36	0.44	2.30	-1.72	down
F7CWC8	oxidase	48.7	59.07	0.96	0.65	0.97	-1.19	-1.11	-0.27	2.28	1.72	up
A0A3Q2LE47	inhibitor heavy chain family member 4	5.6	97.69	0.85	0.82	0.91	-0.69	-0.43	-1.46	2.28	1.72	up
A0A3Q2GWN9	Beta-1 metal-binding globulin	46.2	78.05	0.84	0.90	0.83	-0.48	-0.62	-1.48	2.26	1.71	up
P82187	Kappa-casein	18.4	21.02	0.67	1.38	0.53	-0.80	-0.59	-1.18	2.26	1.71	up
F6V881	Vitronectin	3.6	53.80	0.94	0.87	0.76	-1.28	-0.25	-1.04	2.25	1.71	up
A0A3Q2KK41	member RAS oncogene family	26.8	22.68	-0.92	-1.37	-0.27	0.90	0.86	0.80	2.22	-1.71	down

F6QHY8	intracellular channel protein	13.8	28.73	-0.76	-0.99	-0.81	1.41	0.32	0.83	2.22	-1.71	down
H9GZN9	Uncharacterized protein	31.4	56.92	0.91	1.03	0.63	-0.28	-1.01	-1.28	2.22	1.71	up
A0A3Q2GVU8	Annexin	9.1	38.60	-0.65	-1.48	-0.43	0.78	0.96	0.82	2.19	-1.70	down
A0A3Q2LFU0	inhibitor heavy chain 1	4	96.76	0.95	0.71	0.90	-0.28	-1.40	-0.87	2.17	1.70	up
A0A0A1E4I0	lambda light chain variable region	30.6	22.70	0.78	0.86	0.91	-0.54	-1.51	-0.50	2.16	1.70	up
A0A3Q2KMJ0	Moesin	9.7	63.94	-0.77	-1.14	-0.63	0.40	1.41	0.74	2.15	-1.70	down
A0A3Q2HQW3	tryptophan 5-monooxygenase activation protein beta	14.2	28.11	-1.43	-0.47	-0.64	0.80	1.14	0.60	2.14	-1.70	down
F7CYR1	family C member 1	24.6	56.21	1.26	0.87	0.42	-1.32	-0.58	-0.64	2.13	1.70	up
P13613	Beta-lactoglobulin-1	56.2	18.53	0.87	0.85	0.82	-0.33	-0.72	-1.49	2.11	1.69	up
F6XEB4	3-monooxygenase/tryptophan 5-monooxygenase activation protein zeta	26.4	25.93	-0.83	-1.25	-0.46	0.59	1.36	0.59	2.11	-1.69	down
O77811	Lactotransferrin	55.7	75.99	0.26	1.24	1.03	-0.61	-0.72	-1.20	2.07	1.69	up
F7DQS6	mutase	24.8	28.82	-0.97	-0.22	-1.34	0.68	1.09	0.76	2.06	-1.69	down
A0A3Q2HDB0	associated protein 5	0.4	199.17	0.40	1.53	0.60	-0.75	-0.95	-0.83	2.05	1.68	up
F6USP9	Plasminogen	6.5	91.13	1.43	0.54	0.55	-0.84	-0.50	-1.18	2.04	1.68	up
P17066	shock 70 kDa protein 6	7.6	71.03	-1.36	-0.17	-1.00	0.75	0.89	0.88	2.04	-1.68	down
A0A3Q2HQX4	member RAS oncogene family	10.5	22.70	-1.31	-0.18	-1.04	0.92	0.64	0.96	2.04	-1.68	down
F6QUF7	Lipoprotein lipase	25.1	52.72	-0.22	-1.39	-0.91	1.05	0.65	0.82	2.03	-1.68	down
F6X9U4	Alpha-dystroglycan	3.7	97.25	-0.65	-0.34	-1.53	0.90	0.72	0.89	2.02	-1.68	down
A0A3Q2IDD2	C3/C5 convertase	20.2	85.51	0.88	0.71	0.93	-1.11	-0.13	-1.28	2.00	1.67	up
P19647	Beta-lactoglobulin-2	71.8	18.26	0.90	0.84	0.77	-0.11	-1.15	-1.25	1.99	1.67	up
C3W972	s1 casein	28.3	25.28	0.89	1.22	0.40	-1.06	-1.17	-0.27	1.98	1.67	up
Q5T749	proline-rich protein	5.7	64.14	0.49	1.26	0.75	-0.85	-1.34	-0.32	1.96	1.67	up
F7B5C4	Vimentin	17.2	53.68	-0.30	-1.27	-0.93	0.57	1.31	0.63	1.96	-1.67	down
A0A3Q2HBP1	binding protein 1	14.1	29.11	0.51	1.07	0.93	-0.63	-0.39	-1.48	1.96	1.67	up
A0A3Q2HYP3	phosphoprotein 1	45.3	34.98	0.88	0.81	0.80	-0.13	-0.97	-1.39	1.96	1.67	up
F6VJR6	Alpha-1B-glycoprotein	28	44.90	1.11	1.03	0.35	-0.55	-0.54	-1.41	1.93	1.66	up
P00441	dismutase [Cu-Zn]	7.8	15.94	-0.60	-0.86	-1.04	0.12	1.12	1.25	1.93	-1.66	down
A0A0B4J1B8	domain-binding glutamic acid-rich-like protein	10.8	10.44	-0.22	-1.40	-0.87	0.70	1.17	0.62	1.92	-1.66	down
Q861K5	class I antigen	9.6	30.26	0.76	0.73	0.98	-0.24	-0.70	-1.54	1.88	1.65	up

Q9UNQ0	cassette sub-family G member 2	8.2	72.31	-1.05	-0.82	-0.60	0.82	0.17	1.47	1.84	-1.64	down
A0A3Q2I1H0	kinase C substrate 80K-H	2.3	59.09	-1.29	-0.11	-1.06	0.87	0.51	1.08	1.83	-1.64	down
A0A3Q2HRU9	binding protein	17.8	30.79	0.70	0.91	0.84	-0.63	-0.25	-1.58	1.83	1.64	up
P80303	Nucleobindin-2	17.4	50.22	-1.08	-0.05	-1.34	0.96	0.73	0.77	1.82	-1.64	down
F6ZPY1	domain family member D2	9.7	26.56	-0.14	-0.98	-1.35	0.51	1.14	0.81	1.82	-1.64	down
F7BN14	IFI30 lysosomal thiol reductase	15.9	27.41	0.79	0.92	0.75	-0.83	-0.12	-1.51	1.81	1.64	up
Q13217	homolog subfamily C member 3	17.1	57.58	1.05	1.06	0.35	-0.99	-1.28	-0.18	1.81	1.64	up
F6QAQ9	protein	0.4	207.92	-0.52	-1.31	-0.62	0.44	1.47	0.54	1.78	-1.63	down
F7CG05	Ectonucleotide pyrophosphatase/phosphodiesterase 3	2.7	99.50	-0.23	-1.53	-0.68	0.99	0.95	0.50	1.77	-1.63	down
Q9BXJ0	C1q tumor necrosis factor-related protein 5	6.2	25.30	-0.51	-0.33	-1.60	0.82	0.60	1.02	1.76	-1.63	down
Q9NTX5	decarboxylase	3.6	33.70	-1.01	-0.88	-0.55	0.03	1.18	1.23	1.76	-1.63	down
F6PH25	phosphodiesterase 1	4	91.06	1.09	0.72	0.62	-1.07	-0.04	-1.32	1.76	1.62	up
H9GZV1	Uncharacterized protein	26.5	40.64	0.80	0.84	0.80	0.02	-1.20	-1.25	1.75	1.62	up
A0A3Q2LEZ9	binding lectin serine peptidase 1	4.3	82.88	-0.76	-0.16	-1.51	1.04	0.52	0.88	1.75	-1.62	down
Q01469	acid-binding protein 5	22.2	15.16	-1.30	-0.78	-0.34	0.40	1.44	0.58	1.72	-1.62	down
F6U1J0	Complement factor H	4.4	134.70	0.74	0.87	0.80	-0.99	-1.44	0.02	1.68	1.61	up
Q99816	susceptibility gene 101 protein	4.4	43.94	-1.46	-0.90	-0.05	0.53	1.04	0.84	1.68	-1.61	down
F7AAK7	Actin gamma 1	57.9	41.79	-0.96	-0.96	-0.47	0.47	1.62	0.30	1.63	-1.59	down
F6TVZ7	divalent cation tolerance homolog	6.2	18.79	1.37	0.35	0.67	-0.42	-0.51	-1.45	1.63	1.59	up
P63000	C3 botulinum toxin substrate 1	22.4	21.45	-1.64	-0.44	-0.29	1.01	0.86	0.51	1.61	-1.59	down
A0A3Q2H448	P2 protein	16.4	8.31	-0.01	-1.26	-1.11	0.37	1.11	0.90	1.61	-1.59	down
A0A3Q2I2V3	A1 domain-containing protein	18.1	57.52	0.83	0.70	0.85	-0.11	-1.65	-0.61	1.59	1.58	up
Q9BRK5	kDa calcium-binding protein	11	41.81	0.12	-1.33	-1.16	0.74	0.76	0.87	1.58	-1.58	down
K9K9H6	one binder kinase activator-like 1B-like protein (Fragment)	8.5	15.26	-0.33	-0.95	-1.08	-0.01	1.15	1.23	1.56	-1.57	down

F6ZSB4	alpha chain	2	50.25	-1.01	-0.62	-0.71	0.67	1.63	0.04	1.52	-1.56	down
F7C450	2-HS glycoprotein	23.5	28.34	0.75	0.72	0.87	-1.28	0.18	-1.22	1.50	1.55	up
F7CZW9	family G member 1	3.2	62.28	0.86	0.63	0.84	-0.48	-0.14	-1.71	1.50	1.55	up
F7APU2	Complement factor I	15.6	70.54	0.89	0.70	0.72	-1.46	0.18	-1.02	1.45	1.54	up
F6ZBH7	nucleotide exchange factor	6.7	52.35	-0.64	-1.67	0.00	0.94	0.71	0.65	1.44	-1.54	down
F6PH38	beta chain	14.9	56.25	0.11	0.47	1.72	-0.84	-0.59	-0.86	1.44	1.53	up
P00387	b5 reductase 3	10	34.23	-0.59	-1.18	-0.52	1.20	1.23	-0.13	1.44	-1.53	down
A0A0B4J1C5	A-52 residue ribosomal protein fusion product 1	35.2	14.73	-1.25	-0.51	-0.54	0.73	0.03	1.53	1.43	-1.53	down
K9K4B7	shock 70 kDa protein 13-like protein	19.1	46.51	0.17	1.14	0.98	-0.04	-0.83	-1.42	1.42	1.53	up
K9KGK6	protein 1-like protein	15.1	10.72	-0.27	-0.82	-1.19	1.24	-0.08	1.13	1.42	-1.53	down
F6TIR2	Lipocalin 2	36	33.91	0.38	0.16	1.74	-0.58	-0.92	-0.79	1.41	1.52	up
F6T9W2	and flagella associated protein 157	1.7	73.52	-0.33	-0.84	-1.11	1.32	1.10	-0.14	1.39	-1.52	down
P13796	Plastin-2	20.9	70.29	-0.69	-1.08	-0.51	0.25	1.72	0.30	1.39	-1.52	down
K9K202	division control protein 42-like protein	20.9	21.26	-1.22	-0.58	-0.48	0.45	1.65	0.17	1.39	-1.51	down
O14745	exchange regulatory cofactor NHE-RF1	3.1	38.87	-1.63	-0.76	0.12	0.65	0.74	0.88	1.38	-1.51	down
A0A3Q2HT63	domain-containing protein	2.1	44.43	-0.38	-1.11	-0.78	0.37	1.69	0.20	1.37	-1.51	down
P26583	mobility group protein B2	11.5	24.03	-0.49	-0.29	-1.47	0.24	1.47	0.55	1.35	-1.50	down
F6YY66	diphosphate kinase	19.1	17.26	-0.66	-1.66	0.07	0.66	1.02	0.56	1.34	-1.50	down
F6XWM5	Haptoglobin	38.3	38.47	0.71	0.53	1.00	-0.48	-0.05	-1.72	1.34	1.50	up
F7D8I6	Xanthine dehydrogenase	34.9	146.77	-1.58	-0.82	0.16	1.03	0.54	0.68	1.34	-1.50	down
F6SP02	tryptophan 5-monooxygenase activation protein theta	9.8	27.76	-0.50	-1.50	-0.24	0.29	1.46	0.48	1.32	-1.49	down
