# Supplementary material for: Comparative Whey Proteome Profiling of Donkey Milk With Human and Cow Milk
Source: Front Nutr. 2022 Jun 27;9:911454. doi: 10.3389/fnut.2022.911454 (PMC9282231; doi:10.3389/fnut.2022.911454)
Supplement: Supplementary file 2 [file Data_Sheet_2.PDF]

1.Detailed information of GO-based enrichment of the differentially expressed proteins (DM vs CM)

Category	Term	Count	%	*P* value	Genes	List Total	Pop Hits	Pop Total	Fold Enrichment	FDR
BP	positive regulation of cholesterol esterification	5	2.4038	2.44E-07	F6VBP9, F6Z2L5, F6RM73, F6W4R2, F6RZ27	113	7	12023	75.99873578	7.63E-05
BP	triglyceride catabolic process	5	2.4038	4.78E-06	F6VBP9, F6Z5E1, F6Z2L5, F6QUF7, F6RZ27	113	13	12023	40.92239619	9.95E-04
BP	very-low-density lipoprotein particle remodeling	4	1.9231	2.68E-05	F6VBP9, F6Z2L5, F6QUF7, F6RZ27	113	7	12023	60.79898862	0.002788
BP	high-density lipoprotein particle assembly	4	1.9231	2.68E-05	F6VBP9, F6Z2L5, F6RM73, F6RZ27	113	7	12023	60.79898862	0.002788
BP	cholesterol homeostasis	6	2.8846	7.98E-05	F6VBP9, F6Z5E1, F6Z2L5, F6RM73, F6QUF7, F6RZ27	113	48	12023	13.29977876	0.006225
BP	reverse cholesterol transport	4	1.9231	1.63E-04	F6VBP9, F6Z2L5, F6RM73, F6RZ27	113	12	12023	35.4660767	0.010162
BP	regulation of intestinal cholesterol absorption	3	1.4423	8.44E-04	F6Z2L5, F6RM73, F6RZ27	113	5	12023	63.83893805	0.047905
CC	blood microparticle	20	9.6154	1.39E-23	F7BXD8, F6PQ46, P35747, F6Z2L5, F7C450, F7BFJ1, F7AAK7, F6X1I8, F6RZ27, F7CZW9, F6VBP9, F6RA08, F6RM73, F6W2Y1, F6W4R2, A0A0B4J1C4, F6XEB4, F7CZ92, F6VJR6, F6V881	125	69	13544	31.40637681	8.18E-22
CC	myelin sheath	11	5.2885	3.49E-07	F6Z4J4, F6Z8W0, B3IVM1, P35747, C6L1J5, F7AAK7, K9K202, Q9GKX7, F7B320, F7BFT1, F6ZFH9	125	132	13544	9.029333333	1.03E-05
CC	chylomicron	5	2.4038	4.55E-07	F6VBP9, F6Z2L5, F6RM73, F6QUF7, F6RZ27	125	8	13544	67.72	1.07E-05
CC	very-low-density lipoprotein particle	5	2.4038	1.35E-06	F6Z5E1, F6Z2L5, F6RM73, F6QUF7, F6RZ27	125	10	13544	54.176	2.65E-05
CC	high-density lipoprotein particle	5	2.4038	4.49E-06	F7C0Y4, F6VBP9, F6Z2L5, Q6X9W5, F6RZ27	125	13	13544	41.67384615	6.62E-05
CC	extracellular region	10	4.8077	3.27E-04	F6XWM5, Q6X9X1, F6TIR2, F7CTF0, P02758, F6QUF7, F6W4R2, F6RRV1, Q865P6, F6VJR6	125	237	13544	4.571814346	0.004292
CC	cortical actin cytoskeleton	4	1.9231	0.0023	F6SRP7, F6UME7, F6Y0D9, F7CL80	125	29	13544	14.94510345	0.027142
MF	cholesterol transporter activity	5	2.4038	3.57E-06	F6VBP9, F6Z5E1, F6Z2L5, F6RM73, F6RZ27	108	12	11279	43.51466049	5.90E-04
MF	phosphatidylcholine binding	4	1.9231	2.22E-04	F6Z2L5, F6RM73, A0A0B4J1C4, F6RZ27	108	13	11279	32.13390313	0.012185

2.Detailed information of GO-based enrichment of the differentially expressed proteins (DM vs HM)

Category	Term	Count	%	*P* value	Genes	List Total	Pop Hits	Pop Total	Fold Enrichment	FDR
BP	reverse cholesterol transport	5	3.3557	5.97E-07	F6TN81, F6VBP9, F6Z2L5, F6RM73, F6RZ27	74	12	12023	67.69707207	1.20E-04
BP	high-density lipoprotein particle assembly	4	2.6846	7.39E-06	F6VBP9, F6Z2L5, F6RM73, F6RZ27	74	7	12023	92.84169884	5.92E-04
BP	regulation of cholesterol esterification	4	2.6846	7.39E-06	F6VBP9, F6Z2L5, F6RM73, F6RZ27	74	7	12023	92.84169884	5.92E-04
BP	very-low-density lipoprotein particle remodeling	4	2.6846	7.39E-06	F6VBP9, F6Z2L5, F6QUF7, F6RZ27	74	7	12023	92.84169884	5.92E-04
BP	cholesterol homeostasis	6	4.0268	1.00E-05	F6TN81, F6VBP9, F6Z2L5, F6RM73, F6QUF7, F6RZ27	74	48	12023	20.30912162	6.71E-04
BP	phospholipid efflux	4	2.6846	2.50E-05	F6VBP9, F6Z2L5, F6RM73, F6RZ27	74	10	12023	64.98918919	0.001432
BP	triglyceride catabolic process	4	2.6846	5.88E-05	F6VBP9, F6Z2L5, F6QUF7, F6RZ27	74	13	12023	49.99168399	0.002947
BP	regulation of intestinal cholesterol absorption	3	2.0134	3.59E-04	F6Z2L5, F6RM73, F6RZ27	74	5	12023	97.48378378	0.0131
BP	positive regulation of macrophage cytokine production	3	2.0134	7.49E-04	F7B3U1, F6U904, F7C7V8	74	7	12023	69.63127413	0.023095
BP	lipoprotein metabolic process	3	2.0134	0.001273	F6Z2L5, F6RM73, F6RZ27	74	9	12023	54.15765766	0.034044
CC	blood microparticle	21	14.0940	1.23E-28	F6T0P6, F7BXD8, F6PQ46, P35747, F6Z2L5, F7C450, F7BFJ1, F7AAK7, F6UZH0, F7C0D9, F6X1I8, F6RZ27, F7CZW9, F6VBP9, F6RM73, A0A0B4J1C4, F6XEB4, F7CYR1, F6VJR6, F6PH38, F6V881	88	69	13544	46.84189723	1.21E-26
CC	extracellular space	36	24.1611	1.61E-20	F7BM31, F7APU2, F6W1N4, F6X9U4, F7BPX8, F7B0S3, F7B3U1, F6UME7, F6T7K7, F6WQ61, F6ZBH7, F6VF11, F7CSL8, P82187, F7DXM5, F6RRV1, F6UL68, F7CYR1, F7CAB8, F7C603, F7C0Y4, F6VB94, P35747, F6YNH6, O77811, F7CGP9, F7D8I6, F6ZNX3, F6U904, P11376, F7CZW9, F6TN81, F6WR95, F6PH25, Q6X9W5, F6VUW2	88	825	13544	6.716033058	5.24E-19

CC	chylomicron	5	3.3557	1.09E-07	F6VBP9, F6Z2L5, F6RM73, F6QUF7, F6RZ27	88	8	13544	96.19318182	2.67E-06
CC	high-density lipoprotein particle	5	3.3557	1.09E-06	F7C0Y4, F6VBP9, F6Z2L5, Q6X9W5, F6RZ27	88	13	13544	59.1958042	2.13E-05
CC	focal adhesion	11	7.3826	2.17E-05	F6Z4J4, F6WQ61, F6X9U4, F7AAK7, A5YBL8, K9K202, F6Y0D9, F6XEB4, F6SP02, F7B5C4, F7B821	88	300	13544	5.643333333	3.05E-04
CC	myelin sheath	8	5.3691	2.18E-05	F6Z4J4, F6WQ61, P35747, F7AAK7, K9K202, A0A0B4J1C5, F6YL06, F6ZSB4	88	132	13544	9.327823691	3.05E-04
CC	extracellular region	9	6.0403	1.39E-04	F6XWM5, F6USP9, F6TIR2, P02758, F6QUF7, F6RRV1, Q865P6, F6VJR6, F6PH38	88	237	13544	5.844649022	0.001512
MF	phosphatidylcholine-sterol O-acyltransferase activator activity	4	2.6846	3.27E-06	F6VBP9, F6Z2L5, F6RM73, F6RZ27	80	5	11279	112.79	3.90E-04
MF	serine-type endopeptidase inhibitor activity	6	4.0268	5.99E-05	F7CZW9, F7BM31, F7CSL8, F7DXM5, F6UZH0, F7CYR1	80	60	11279	14.09875	0.002675
MF	cholesterol transporter activity	4	2.6846	6.95E-05	F6VBP9, F6Z2L5, F6RM73, F6RZ27	80	12	11279	46.99583333	0.002675

3.Detailed information of KEGG pathway-based enrichment of the differentially expressed proteins (DM vs CM)

KEGG pathway	Count	%	*P* value	Genes	List Total	Pop Hits	Pop Total	Fold Enrichment	FDR
Antigen processing and presentation	8	3.8462	7.62E-06	Q9GKX8, Q6X9X1, F7BPX8, F6YNH6, Q9GKX7, F7BN14, F6VUW2, F6TQR2	81	64	6936	10.70	0.0010367
Glycolysis / Gluconeogenesis	7	3.3654	5.40E-05	B3IVM1, C6L1J5, F7D1R1, F6X8Q2, P00559, F6SX98, F7DQS6, F6TZS9	81	59	6936	10.16	0.0036686
Complement and coagulation cascades	7	3.3654	1.31E-04	F7CZW9, F7BM31, F7APU2, F7BFJ1, F7DXM5, F6W2Y1, F6XSF7	81	69	6936	8.69	0.0059379
Lysosome	8	3.8462	3.54E-04	F7BFV9, F6V6F7, F7BPX8, F6YNH6, F7BMS8, F7DG10, F6PH25, F6VUW2	81	116	6936	5.91	0.0120341
Biosynthesis of antibiotics	9	4.3269	0.0023729	B3IVM1, C6L1J5, F7D1R1, F6X8Q2, F6Y4I1, P00559, F6SX98, F7CL92, F7DQS6, F6TZS9	81	205	6936	3.76	0.0645431
Phagosome	7	3.3654	0.0070083	Q95M34, Q5XWB8, F7AAK7, F7BPX8, F7DG10, F6VUW2, F6TQR2	81	148	6936	4.05	0.1485799

4.Detailed information of KEGG pathway-based enrichment of the differentially expressed proteins (DM vs HM)

KEGG pathway	Count	%	*P* value	Genes	List Total	Pop Hits	Pop Total	Fold Enrichment	FDR
Complement and coagulation cascades	9	6.0403	2.87E-08	F7CZW9, F6USP9, F7BM31, F7APU2, F7BFJ1, F7DXM5, F7C0D9, F7CYR1, F6PH38	52	69	6936	17.39799331	3.47E-06
Phagosome	7	4.6980	6.96E-04	Q95M34, Q5XWB8, F7AAK7, F7BPX8, F6U904, F6ZSB4, F6VUW2	52	148	6936	6.308731809	0.042102
Antigen processing and presentation	4	2.6846	0.011388	F7BPX8, F6YNH6, F7BN14, F6VUW2	52	64	6936	8.336538462	0.374498
PPAR signaling pathway	4	2.6846	0.01238	F6Z2L5, F6RM73, F6QUF7, F6U904	52	66	6936	8.083916084	0.374498
Fat digestion and absorption	3	2.0134	0.033037	F6Z2L5, F6U904, F6RZ27	52	39	6936	10.26035503	0.79949
